# The underlying microbial mechanism of epizootic rabbit enteropathy triggered by a low fiber diet

**DOI:** 10.1038/s41598-018-30178-2

**Published:** 2018-08-21

**Authors:** Ding Xing Jin, Hua Wei Zou, Si Qiang Liu, Li Zhi Wang, Bai Xue, De Wu, Gang Tian, Jingyi Cai, Tian Hai Yan, Zhi Sheng Wang, Quan Hui Peng

**Affiliations:** 1 Institute of Animal Nutrition, Key Laboratory of Bovine Low-Carbon Farming and Safe Production, Sichuan Agricultural University, Ya’an, Sichuan 625014 PR China; 2 Agri-Food and Biosciences Institute, Hillsborough, Co. Down BT26 6DR United Kingdom

## Abstract

Epizootic rabbit enteropathy (ERE) is reproduced successfully in the present study by feeding rabbits a low-fibre diet, and high-throughput sequencing and quantitative real-time PCR (qPCR) analysis were applied to examine the microbial variations in the stomach, small intestine and caecum. The evenness was disturbed and the richness was decreased in the ERE groups. When the rabbits were suffering from ERE, the abundance of the Firmicutes was decreased in three parts of the digestive tract, whereas the Proteobacteria was increased in the stomach and caecum, the Bacteroidetes and Verrucomicrobia were increased in the small intestine. Correlation analysis showed that the reduced concentrations of TVFA and butyrate in the caeca of the ERE group were attributed to the decreased abundances of genera such as *Lactobacillus*, *Alistipes* and other fibrolytic bacteria and butyrate- producing bacteria such as *Eubacterium* and *Faecalibacterium*. It is concluded that, in terms of microorganisms, the overgrowth of *Bacteroides fragilis*, *Clostridium perfringen*, *Enterobacter sakazakii* and *Akkermansia muciniphila* and inhibition of *Bifidobacterium* spp. and *Butyrivibrio fibrisolvens* in the stomach, small intestine and caecum resulted in a decrease in butyrate yield, leading to the incidence of ERE, and the probability of developing ERE could be manipulated by adjusting the dietary fibre level.

## Introduction

Epizootic rabbit enteropathy (ERE) is a severe gastrointestinal syndrome disease that mainly affects post-weaning rabbits and has caused substantial economic losses in rabbit farming regions in France since 1997^1,2^. The morbidity of rabbits after weaning can reach 100%, and the mortality can reach 30–80% in the absence of medication^3^. The first detectable sign of ERE syndrome is a sudden onset of anorexia and a decrease in water consumption, followed by depression and abdominal distension, with alternating constipation and limited liquid diarrhoea, and no obvious lesions or areas of inflammation are observed in any part of the intestine or organs^4^. Although many works have been devoted to identifying the pathogenic microorganism responsible for ERE^5,6^, the aetiology is still incompletely understood. It is noteworthy that ERE was successfully reproduced by inoculating the intestinal contents of sick rabbits, indicating that this disease is infectious^3^, and that ERE was a bacterial disease, and a viral or parasitic aetiology was eliminated^6^. A later study confirmed an infectious aetiology and a bacterial origin by examining histopathological and ultrastructural lesions of intestines^7^. Bacteriological examinations found that the bacteria *Clostridium perfringens* and *Escherichia coli* (*E*. *coli*) were frequently detected in small intestine content or faecal samples from rabbits with ERE in field cases^5,8^.

Fibre is important for rabbits, as the morbidity of ERE rabbits significantly increased after feeding with a fibre- deficient diet^9,10^ or a diet with a low digestible fibre (hemicelluloses + pectins) to starch ratio^11^. Researchers claimed that a low-fiber diet may be a facilitating factor and a possible vector of this disease if not a primary factor^12^. A diet low in fibre slows down the digestive transit and changes the caecal motility pattern, resulting in an impairment of caecal microbial activity and reduction in the level of fibrolytic bacteria^13^. Furthermore, a low level of fibre in the diet is generally accompanied by high a level of starch, which would lead to carbohydrate overload in the hindgut and promote the proliferation of pathogens such as *Clostridium* spp. and *E*. *coli*^14^, ultimately favouring the incidence of enteropathy.

The volatile fatty acids (VFAs) produced in the caecum provide 12~40% of the maintenance energy for rabbits. In addition, VFAs have a profound effect on other host physiological functions, such as the inflammatory response, cell signalling of intestinal epithelial cells, colon mucosal growth, and pathogen protection^15^. Therefore, the relationship between the caecal VFA concentration and microbial composition deserves in-depth investigation. Comparative analysis of the caecal bacterial community showed marked differences between healthy and ERE rabbits^16^. Because ERE could be fully reproduced by inoculation with intestinal contents (including from the small intestine, caecum and colon), microbes from the whole gastrointestinal tract are critical for rabbits health. Hence, examination of the microbes in the gastrointestinal tract of ERE rabbits may facilitate the detection of pathogenic bacteria and the comprehension of the aetiopathogenesis of ERE. This experiment aimed to determine what bacteria might be responsible for the occurrence of ERE by comparing the microbial composition in the stomach, small intestine and caecum between healthy and ERE rabbits. The ERE model was established by feeding rabbits a low-fibre diet. Relationships between microbial genera and fermentation parameters including the VFA molar proportion, were also identified.

## Results

### Analysis of Illumina MiSeq sequencing data from normal and ERE rabbits

Illumina MiSeq sequencing of 16S rRNA yielded 3146039 valid sequences from 36 samples (6 normal rabbits and 6 ERE rabbits; samples were taken from the stomach, small intestine and caecum of each rabbit), with a mean of 87390 sequences per sample. These sequences were clustered into 89988 operational taxonomic units (OTUs) at the 97% similarity level, with an average of 2450 OTUs per sample. Rarefaction, Chao1 and Shannon index curves are shown in Fig. S1. All of the curves asymptotically approached a plateau, suggesting that new phylotypes would not be detected even if additional sequencing was performed and most species were captured. The valid sequences, OTUs, diversity and species richness as estimated using the Shannon, Simpson, Chao1, and Ace indices are shown in Table 1. The α-diversity indices including the Chao1 and Ace indices in caecal samples of normal groups (NC) were significantly greater (*P* < 0.01) than those in stomach, small intestinal samples of normal groups (NS, NI; respectively), and caecal samples of ERE groups (EC) was higher (*P* < 0.01) than those in stomach and small intestinal samples of ERE groups (ES, EI; respectively) (Table 1). No significant differences were observed in the Shannon and Simpson indices among the three parts of the digestive tract in the normal groups (*P* > 0.05); however, EI had lower Simpson indices than ES and EI. On the other hand, the three digestive tract parts of ERE rabbits (ES, EI and EC) had lower bacterial diversity than those in the normal groups (NS, NI and NC). The overall OTU number, species richness indices (Chao1, Ace index), and diversity index (Shannon index) in ERE rabbits were significantly lower (*P* < 0.01) than those in normal rabbits (Table 1). These results indicated that the bacteria in the caecum had higher phylogenetic richness than the bacteria in the stomach and small intestine, and the ERE rabbits had lower bacterial diversity and species richness than normal rabbits.

**Table 1:** Summary of the Illumina MiSeq sequence data and statistical analysis of the bacterial diversity in the stomach, intestine and caecum of normal and ERE rabbits.

Item^1^	S			I			C			*P-*value	
	NS	ES	*P*	NI	EI	*P*	NC	EC	*P*	Normal	ERE
Valid sequence	55920^c^	55675^C^	0.98	92169^b^	69021^B^	0.10	134172^a^	117384^A^	0.23	<0.01	<0.01
OTUs	1263	1055^C^	<0.01	1256	909^B^	<0.01	1303	1211^A^	0.01	0.40	0.02
Chao1	1957^b^	1711^B^	<0.01	1934^b^	1599^B^	<0.01	2152^a^	1914^A^	<0.01	<0.01	<0.01
Ace	2087^b^	1784^B^	<0.01	2047^b^	1715^B^	<0.01	2252^a^	2043^A^	<0.01	<0.01	<0.01
Shannon	5.46	4.86^A^	<0.01	5.32	4.39^B^	<0.01	5.49	5.32^A^	0.02	0.92	0.03
Simpson	0.97	0.95	0.36	0.94	0.93	0.86	0.97	0.97	0.78	0.55	0.07

^1^Data are presented as the mean of six samples, means with different superscripts of minuscule represent significant differences among the three digestive parts of normal rabbits, and means with different superscripts of majuscule represent significant differences among the three parts of ERE rabbits.

**Figure 1:**
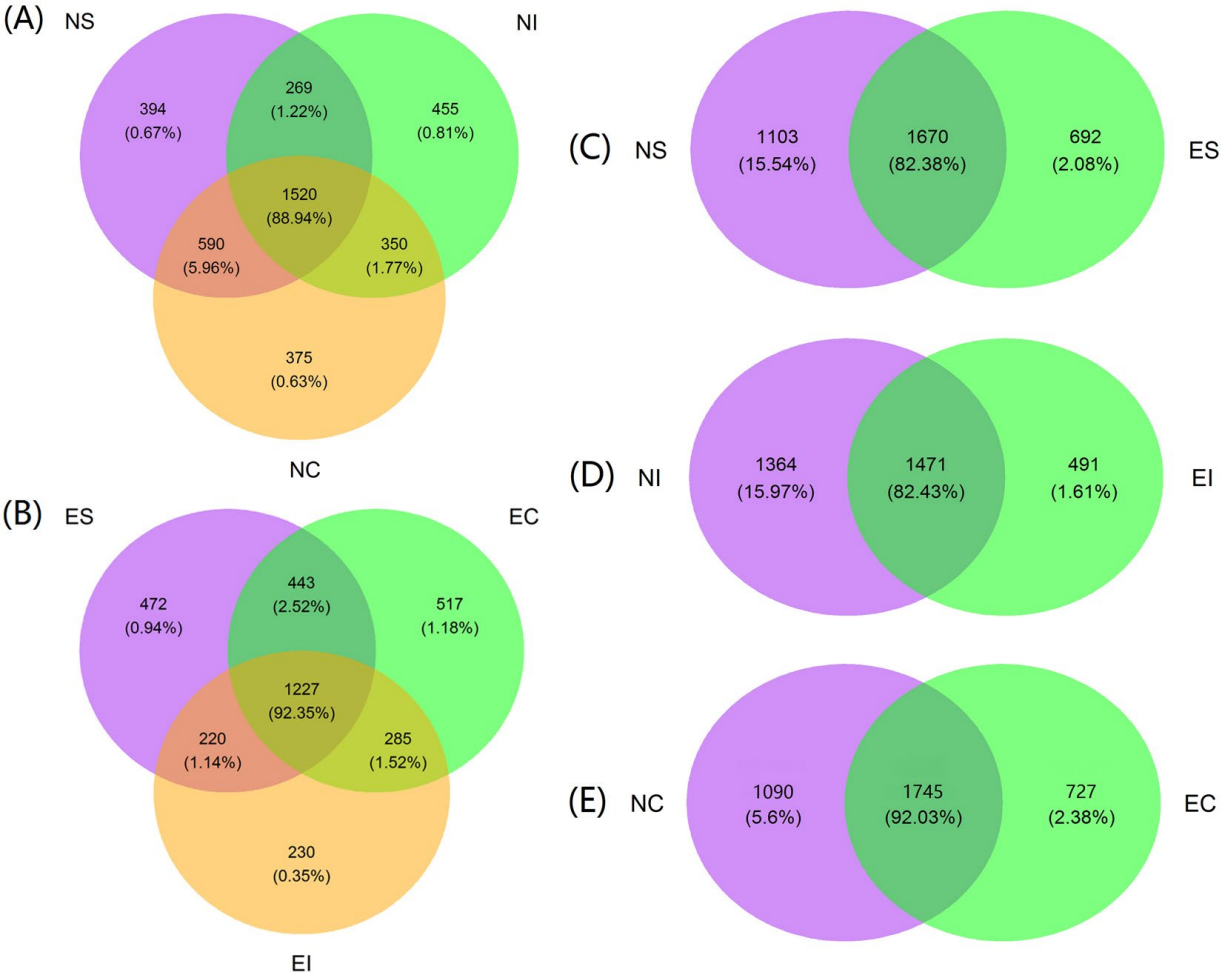
Venn diagram representation of the shared and exclusive OTUs at the 97% similarity level among the three parts of the digestive tracts in normal rabbits and ERE rabbits: between NS and ES, NI and EI and NC and EC.

To better understand the differences between normal and ERE rabbits, the shared and exclusive OTUs (sequences) were visualized using a Venn diagram (Fig. 1). According to the Venn diagram at a 97% similarity level, 1520 and 1227 OTUs (88.94% and 92.35% of the total sequences) were common to normal and ERE rabbits in the three different parts of the digestive tract, respectively. In normal rabbits, the stomach, small intestine and caecum shared 269 (1.22% of sequences) and 590 (5.96% of sequences) OTUs, and the small intestine shared 350 OTUs (1.77% of sequences) with the caecum (Fig. 1A). In parallel, the stomach, small intestine and caecum shared 220 (2.52% of sequences) and 443 (1.14% of sequences) OTUs, and the small intestine shared 285 OTUs (1.52% of sequences) with the caecum in ERE rabbits (Fig. 1B). Compared with the normal rabbits, the OTUs (sequences) unique to ERE rabbits in ES, EI and EC were 692 (2.08%), 491 (1.61%) and 727 (2.38%), respectively (Fig. 1C–E).

**Figure 2:**
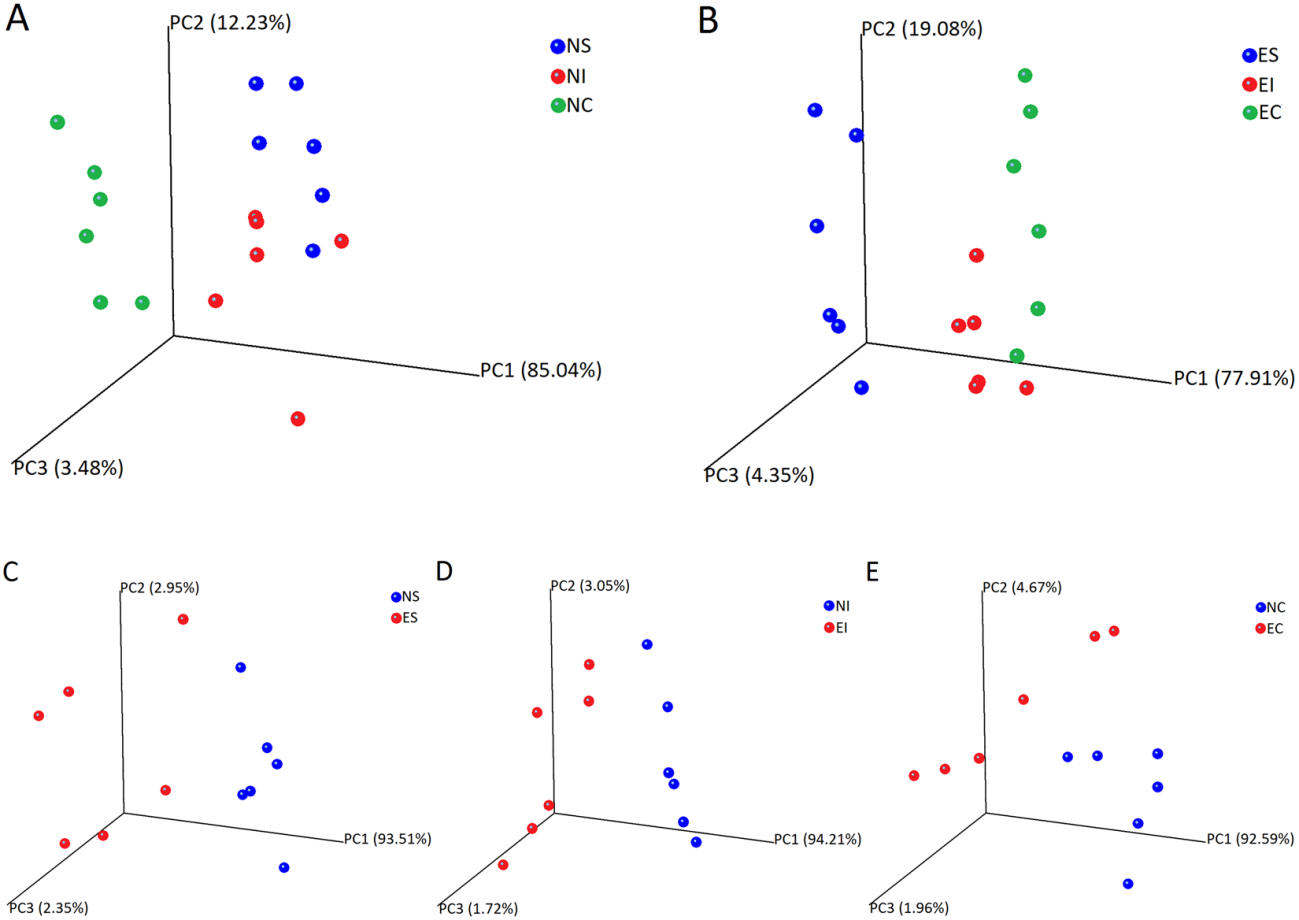
Principal coordinate analysis (PCoA) 3D plot generated based on the Euclidean distance showing distinct differences in the structures of the bacterial community in the three parts of the digestive tract in normal (**A**) and ERE rabbits (**B**) and between NS and ES (**C**), NI and EI (**D**) and NC and EC (**E**).

Principal coordinate analysis (PCoA) was conducted based on the Euclidean distances between the three normal and ERE rabbits and among the three different digestive parts, respectively, and the results are shown in Fig. 2. In both normal or ERE rabbits, the six replicates from the same organ clustered together and could be isolated clearly from the others. These results showed strong differences in the microbial structure among the three parts of the digestive tract (Fig. 2A,B). Another characteristic of the PCoA was that the six replicates of NC and NI were closely clustered, while the six replicates of NC were isolated; however, the replicates of EI and EC were much more closely clustered together than they were with the replicates of ES (Fig. 2B). This might suggest that the stomach and small intestine in normal rabbits had similar microbial compositions, whereas the small intestine and caecum had similar microbial compositions after rabbits became affected by ERE.

The PCoA plot also displayed significant differences in the microbial community in the stomach, small intestine and caecum between normal and ERE rabbits. The three groups of ERE rabbits (ES, EI and EC) could be isolated clearly from the groups of normal rabbits (NS, NI and NC) (PC1 accounted for 93.51% of the variance between NS and ES, 94.21% between NI and EI, and 92.59% between NC and EC). The six replicates from the stomach, small intestine and caecum of ERE rabbits were much more scattered than those of the normal rabbits, impliying that the microbial community ERE rabbits experienced remarkable dysbiosis (Fig. 2C–E). However, from a morphological point of view, the six replicates of NI were even more scattered than the replicates of EI, which might suggest that the microbial composition itself is more variable in the small intestine than in the stomach and caecum.

### Bacterial diversity and composition in the different parts of the gastrointestinal tract in normal and ERE rabbits

**Figure 3:**
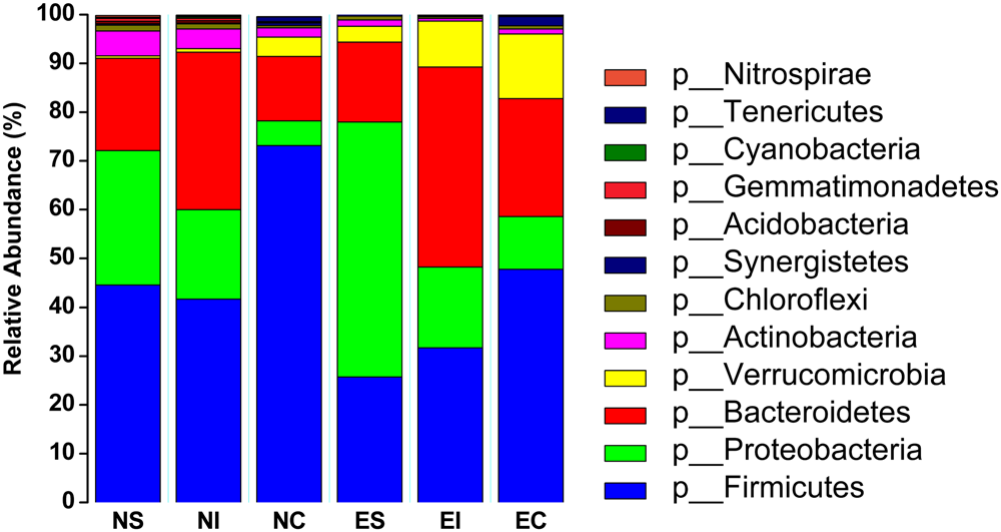
The relative abundance and microbial composition of the dominant phyla in the stomach, small intestine and caecum of normal and ERE rabbits. Each bar represents the average relative abundance of each bacterial taxon within a group. The top 12 phyla are marked on the bar.

In this study, we used the Greengenes database for taxonomic assignment. The most common phyla in the three digestive parts of normal rabbits were Firmicutes (44.60%, 41.69% and 72.12% of the total sequence reads in NS, NI and NC, respectively), Proteobacteria (27.50%, 18.28% and 5.09%), Bacteroidetes (18.93%, 32.33% and 13.23%) and Actinobacteria (5.10% vs. 4.05% and 2.89%) (Fig. 3). Detailed classification information and relative abundances of bacteria are shown in Table S1. In ERE rabbits, the dominant four phyla in the stomach were Proteobacteria (52.25%), Firmicutes (25.73%), Bacteroidetes (16.39%) and Actinobacteria (3.22%). In the small intestine, they were Bacteroidetes (41.06%), Firmicutes (31.71%), Proteobacteria (16.48%) and Actinobacteria (9.42%), and in the caecum, they were Firmicutes (47.72%), Bacteroidetes (24.20%), Verrucomicrobia (13.27%) and Proteobacteria (10.86%) (Fig. 3).

**Figure 4:**
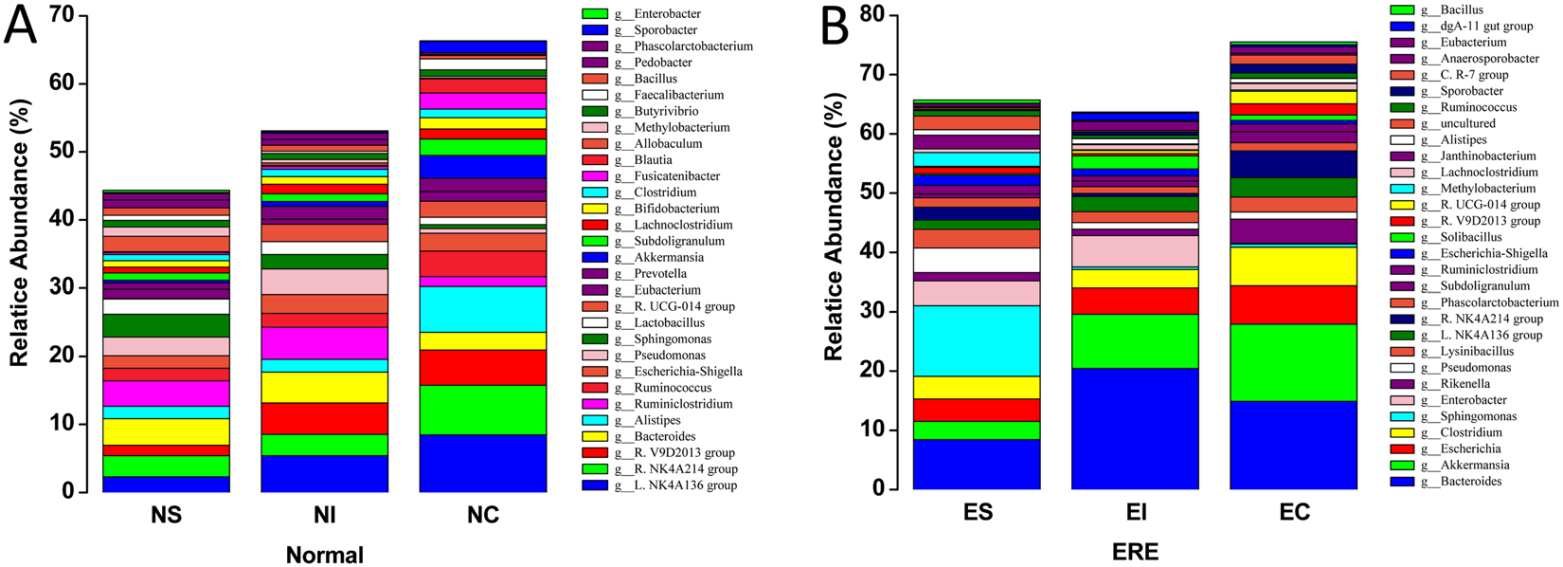
The relative abundance and microbial composition of the dominant genera in the stomach, small intestine and caecum of normal (**A**) and ERE (**B**) rabbits. Each bar represents the average relative abundance of each bacterial taxon within a group. The top 30 genera are marked on the bar.

At a lower taxonomical level, we detected 63 (relative abundance ≥ 0.05%) different genera in the stomach and small intestine contents of healthy rabbits and 61 (the genera *Comamonas* and *Desulfuromonas* were not detected or had a the relative abundance <0.05%) in the caecal contents of healthy rabbits (Table S1). The microbial composition in the three digestive parts of normal rabbits was not exactly the same. In the stomach, the predominant bacterial genera (relative abundance ≥1%) were *Ruminiclostridium* (3.74%), *Bacteroides* (3.93%), *Sphingomonas* (3.37%), *Ruminococcaceae* NK4A214 group (3.10%), *Pseudomonas* (2.71%), *Lachnospiraceae* NK4A136 group (2.30%), *Allobaculum* (2.29%), Lactobacillus (2.24%), *Ruminococcus* (1.85%) and *Alistipes* (1.85%) (Fig. 4A, Table S1). In the small intestine, they were *Lachnospiraceae* NK4A136 group (5.40%), *Ruminiclostridium* (4.73%), *Ruminococcaceae* V9D2013 group (4.61%), *Bacteroides* (4.52%), *Pseudomonas* (3.69%), *Ruminococcaceae* NK4A214 group (3.14%), *Escherichia-Shigella* (2.78%), *Ruminococcaceae* UCG-014 group (2.54%), *Sphingomonas* (2.12%) and *Ruminococcus* (2.03%), and in the caecum, they were *Lachnospiraceae* NK4A136 group (8.46%), *Ruminococcaceae* NK4A214 group (7.27%), *Ruminococcaceae* V9D2013 group (5.20%), *Alistipes* (3.84%), *Ruminococcus* (3.77%), *Akkermansia* (3.36%), *Escherichia-Shigella* (2.62%), *Bacteroides* (2.60%), *Subdoligranulum* (2.41%) and *Ruminococcaceae* UCG-014 group (2.41%) (Fig. 4A, Table S1). In ERE rabbits, we also detected 58 different genera in ES and EC and 56 in EI (genera *Faecalibacterium* and *Brevundimonas* were not detected). The dominant genera in the stomach were *Sphingomonas* (11.93%), *Bacteroides* (8.42%), *Pseudomonas* (4.17%), *Enterobacter* (4.12%), *Clostridium* (3.83%), *Escherichia* (3.80%), *Lysinibacillus* (3.16%), *Akkermansia* (3.08%), *Methylobacterium* (2.32%) and *Janthinobacterium* (2.30%). In the small intestine, they were *Bacteroides* (20.45%), *Akkermansia* (9.12%), *Enterobacter* (5.30%), *Escherichia* (4.39%), *Clostridium* (3.12%), *Lachnospiraceae* NK4A136 group (2.54%), *Solibacillus* (2.22%), *Lysinibacillus* (1.87%), *Anaerosporobacter* (1.53%) and *Escherichia-Shigella* (1.18%). In the caecum, they were *Bacteroides* (14.88%), *Akkermansia* (13.06%), *Clostridium* (6.53%), *Escherichia* (6.39%), *Ruminococcaceae* NK4A214 group (4.55%), *Rikenella* (4.11%), *Lachnospiraceae* NK4A136 group (3.26%), Lysinibacillus (2.51%), *Ruminococcaceae* UCG-014 group (2.13%) and *Ruminococcaceae* V9D2013 group (1.90%) (Fig. 4B, Table S1).

### Impact of ERE on the microbial community in stomach, small intestine and caecum

**Figure 5:**
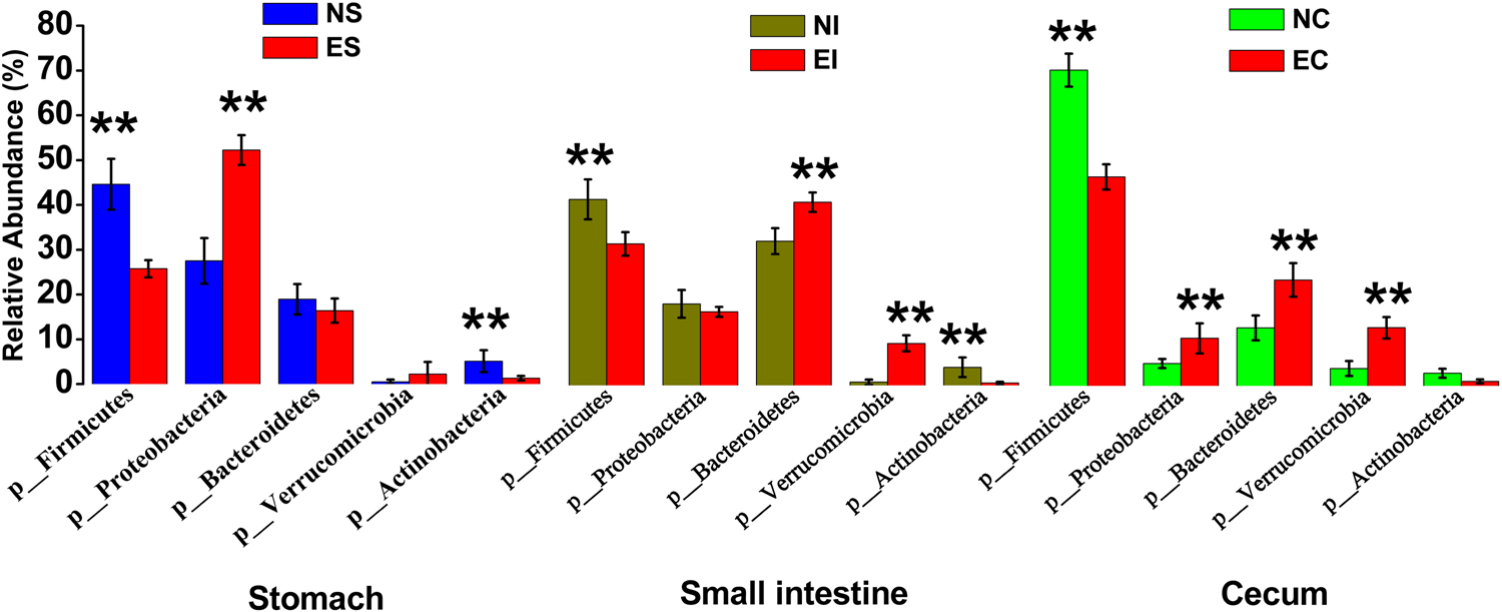
Effect of ERE on the relative abundances of the dominant phyla in the stomach, intestinal and caecal microbiomes of rabbits. Error bars represent the SD of three samples. Asterisks show significant differences between groups (*P < 0.05, **P < 0.01, Mann-Whitney U test).

The bacterial composition in digestive samples was significantly changed after rabbits were affected by ERE. At the phylum level in the stomach, when the rabbits were suffering from ERE, the abundance of Firmicutes (*P* < 0.01) and Actinobacteria (*P* < 0.01) were decreased, and that of Proteobacteria (*P* < 0.01) was increased. In the small intestine, the abundance of Firmicutes (*P* < 0.01) and Actinobacteria (*P* < 0.01) were decreased, while those of Bacteroidetes (*P* < 0.01) and Verrucomicrobia (*P* < 0.01) were increased. In the caecum, the abundance of Firmicutes (*P* < 0.01) was decreased, whereas those of Proteobacteria (*P* < 0.01), Bacteroidetes (*P* < 0.01) and Verrucomicrobia (*P* < 0.01) were increased (Fig. 5, Table S1).

**Figure 6:**
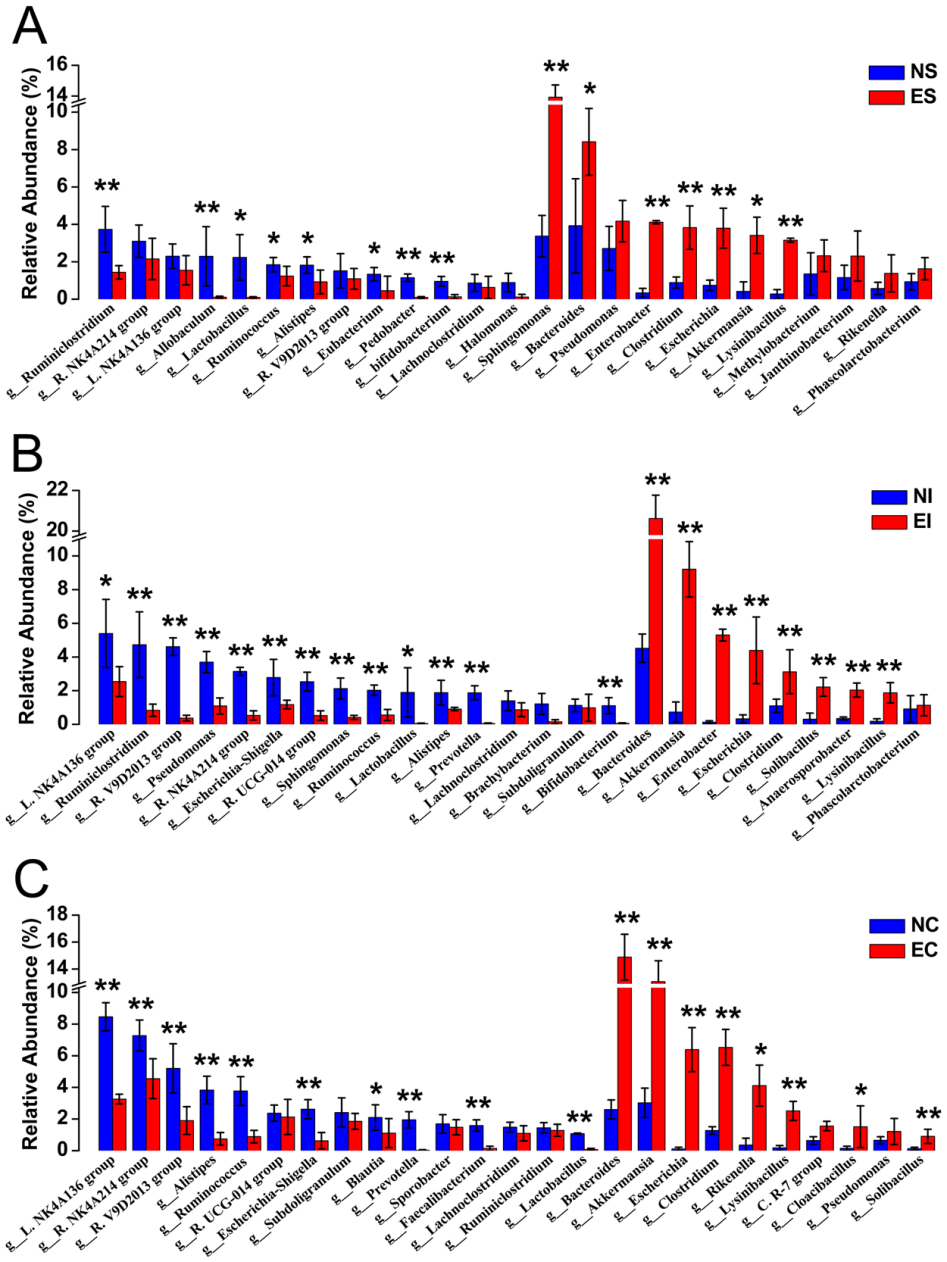
Effect of ERE on the relative abundances of the dominant genera in the stomach (**A**), intestinal (**B**) and caecal (**C**) microbiomes of rabbit. The top 25 dominant genera in each group are shown in this figure. Error bars represent the SD of six samples. Asterisks show significant differences between groups (*P < 0.05, **P < 0.01, Mann-Whitney U test).

At a lower taxonomical level, the abundance of the genera *Ruminiclostridium*, *Allobaculum*, *Lactobacillus*, *Ruminococcus*, *Alistipes*, *Eubacterium*, *Pedobacter* and *Bifidobacterium* in ES were significantly decreased compared with those in NS (*P* < 0.05), and the abundance of the dominant genera in ES (*Sphingomonas*, *Bacteroides*, *Escherichia*, *Enterobacter*, *Clostridium*, *Akkermansia*, *and Lysinibacillus*) were significantly increased compared with those in NS (*P* < 0.05) (Fig. 6A). The abundance of the genera *Lachnospiraceae* NK4A136 group, *Ruminiclostridium*, *Ruminococcaceae* V9D2013 group, *Pseudomonas*, *Ruminococcaceae* NK4A214 group, *Escherichia-Shigella*, *R*.*UCG-014 group*, *Sphingomonas*, *Ruminococcus*, *Lactobacillus*, *Alistipes*, *Prevotella* and *Bifidobacterium* were decreased (*P* < 0.05), while those of the genera *Bacteroides*, *Akkermansia*, *Enterobacter*, *Escherichia*, *Clostridium*, *Solibacillus*, *Anaerosporobacter* and *Lysinibacillus* were increased compared with NS (*P* < 0.05) (Fig. 6B). The genera *Lachnospiraceae* NK4A136 group, *Ruminococcaceae* NK4A214 group, *Ruminococcaceae* V9D2013 group, *Alistipes*, *Ruminococcus*, *Escherichia-Shigella*, *Blautia*, *Prevotella*, *Faecalibacterium* and *Lactobacillus* were decreased (*P* < 0.05) in the caecum of ERE rabbits, while those of the genera *Bacteroides*, *Akkermansia*, *Escherichia*, *Clostridium*, *Rikenella*, *Lysinibacillus*, *Cloacibacillus and Solibacillus* were increased compared with those in NC (*P* < 0.05) (Fig. 6C).

**Figure 7:**
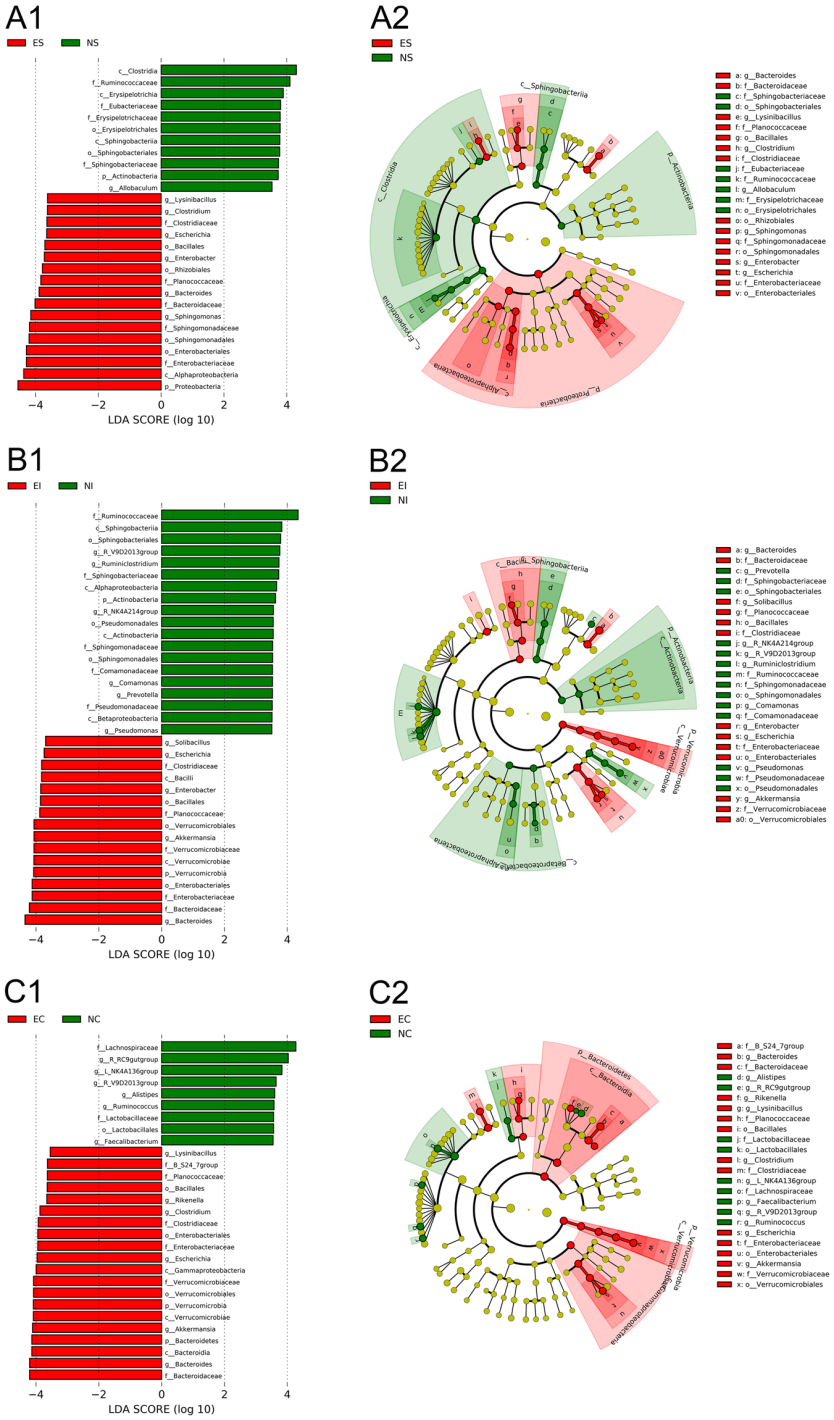
LEfSe identified the most differentially abundant taxa between NS and ES (**A1**,**A2**), NI and EI (**B1**,**B2**), NC and EC (**C1**,**C2**). Only taxa meeting an LDA significance threshold >3.5 are shown.

The linear discriminant analysis (LDA) effect size (LEfSe) method was applied to identify the specific bacterial taxa associated with ERE infection (Fig. 7). A cladogram presents the structure of the microbiota; the predominant bacteria in the stomach, small intestine and caecum were shown in Fig. 7A–C; and the greatest differences in taxa between the three groups are displayed. The results indicated that six genera (*Bacteroides*, *Lysinibacillus*, *Clostridium*, *Escherichia*, *Enterobacter* and *Sphingomoas*) belonging to five families (*Bacteroidaceae*, *Planococcaceae*, *Clostridiaceae*, *Enterobacteriaceae* and *Sphingomonadaceae*) had higher abundances in ES than in NS (*P* < 0.01). Four genera (*Solibacillus*, *Akkermansia*, *Escherichia* and *Enterobacter*) belonging to three families (*Planococcaceae*, *Verrucomicrobiaceae* and *Enterobacteriaceae*), the genus *Bacteroides* and the families *Bacteroidaceae* and *Clostridiaceae* were significantly higher in EI than in NI (*P* < 0.01). Four genera (*Bacteroides*, *Rikenella*, *Akkermansia* and *Escherichia*) belonging to three families (*Bacteroidaceae*, *Verrucomicrobiaceae* and *Enterobacteriaceae*) and two phyla (Bacteroidetes and Verrucomicrobia), and two genera *Lysinibacillus* and *Clostridium* belonging to two families (*Planococcaceae* and *Clostridiaceae*) were also significantly higher in EC than in NC (*P* < 0.01).

**Figure 8:**
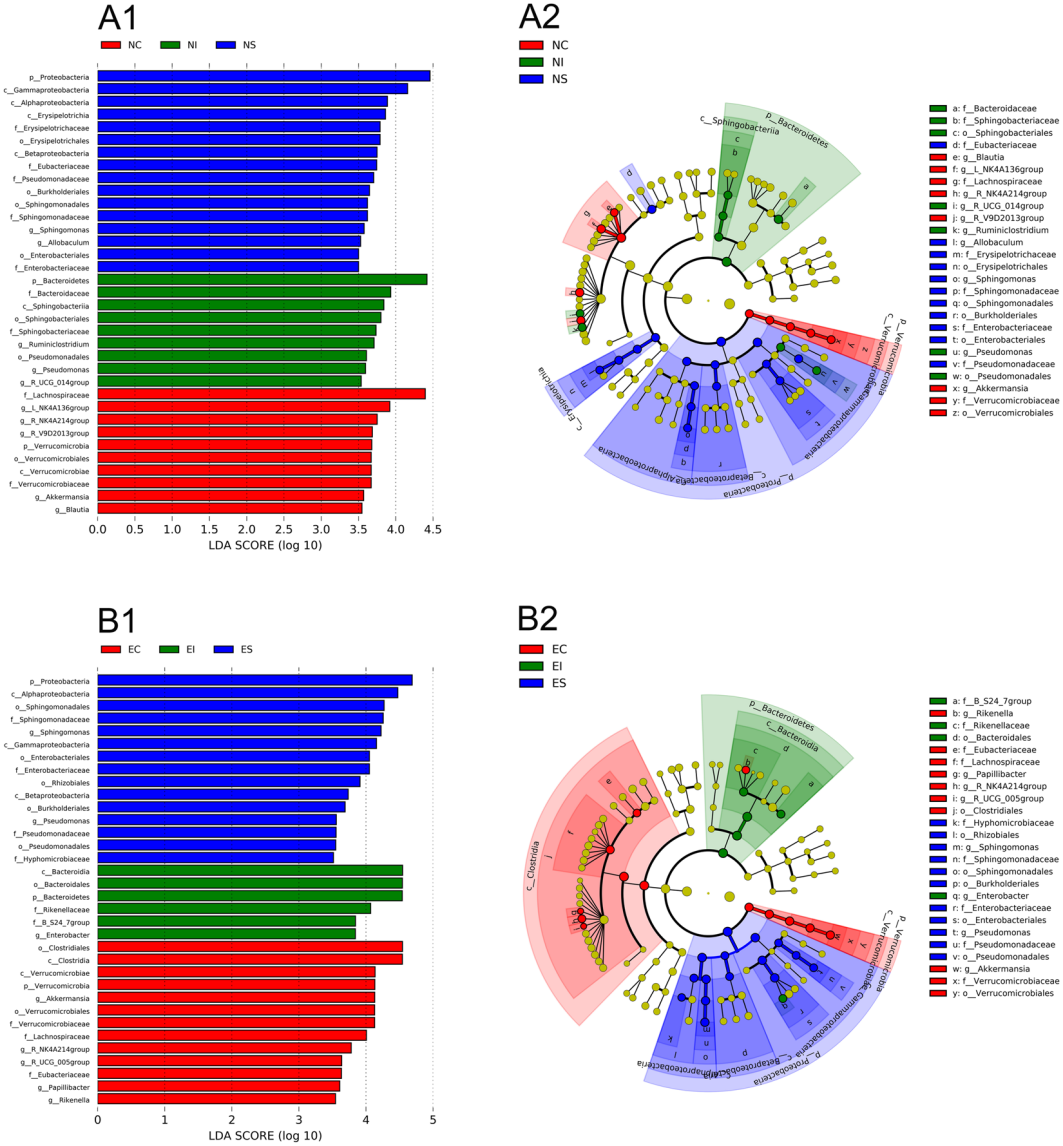
LEfSe identified the most differentially abundant taxa among the three parts of the digestive tract in normal rabbits (**A1**,**A2**) and in ERE rabbits (**B1**,**B2**). Only taxa meeting an LDA significance threshold >3.5 are shown.

In addition, to identify differences in the microbial composition in the stomach, small intestine and caecum, the microbiota in the three normal groups and three ERE groups were also compared (Fig. 8). Eleven specific bacterial taxa (e.g., *Eubacteriaceae*, *Allobaculum*, *Erysipelotrichaceae*, *Erysipelotrichales* and *Sphingomonas*) were significantly more abundant in ERE group (LDA > 3.5) than in NS (Fig. 8A), and there were 6 and 8 specific bacterial taxa in NI and NC, respectively. The results also show that the stomach and caecum had more specific bacterial taxa (11 and 10, respectively) related to ERE than the small intestine (Fig. 8B).

Finally, qPCR was performed to analyse the abundance of certain species and to confirm significant differences in the proportion of specific taxonomic groups between the ERE and normal groups (Table S2). As indicated by the qPCR results, the total bacterial abundance in the caecal contents was higher than that in the stomach and small intestine contents (NC = 9.89, NS = 5.78, NI = 7.59; log_10_^copy number/g content^). On the other hand, the copy numbers of the total bacteria in the three parts of ERE rabbits were significantly decreased compared with that of normal rabbits (*P* < 0.05).

In the stomach, the copy numbers of *Sphingomonas paucimobilis*, *Bacteroides fragilis*, *Bacteroides thetaiotaomicron* and *Clostridium perfringens* in ES were increased by more than 1 log unit (ten times) compared with those in NS (*P* < 0.05), but the copy numbers of *Lactobacillus* group, *Bifidobacterium* group and *Butyrivibrio fibrisolvens* in ES were decreased by more than 1 log unit compared with those in NS (*P* < 0.05). In the small intestine, the copy numbers of *Clostridium perfringens*, *Enterobacter sakazakii*, *Akkermansia muciniphila*, *Bacteroides fragilis*, *Escherichia coli* and *Bacteroides caccae* in EI were increased by more than 1 log unit compared with those in NI (*P* < 0.05); however, the copy numbers of *Bifidobacterium* group, *Butyrivibrio fibrisolvens*, *Bifidobacterium* group, *Faecalibacterium prausnitzii*, *Eubacterium* group and *Lactobacillus* group were decreased by more than 1 log unit compared with those in NI (*P* < 0.05). In the caecum, the copy numbers of *Clostridium perfringens*, *Bacteroides fragilis*, *Clostridium difficile*, *Bacteroides ovatus*, *Bacteroides stercoris*, *Bacteroides thetaiotaomicron* and *Bacteroides vulgatus* in EC were increased by more than ten times compared with those in NC (*P* < 0.05). *Escherichia coli* and *Akkermansia muciniphila* increased more than a hundred times (2 log units) form 6.05 log_10_^copy number/g content^ to 8.18 and from 6.22 to 8.39 compared with NC (*P* < 0.05). In contrast, the copy numbers of *Ruminococcus albus*, *Eubacterium group*, *Alistipes* group, *Faecalibacterium prausnitzii*, *Bifidobacterium* group and *Ruminococcus flavefaciens* in EC were decreased by more than ten times compared with those in NC (*P* < 0.05).

### Impact of ERE on the caecal pH and volatile fatty acid concentration

**Figure 9:**
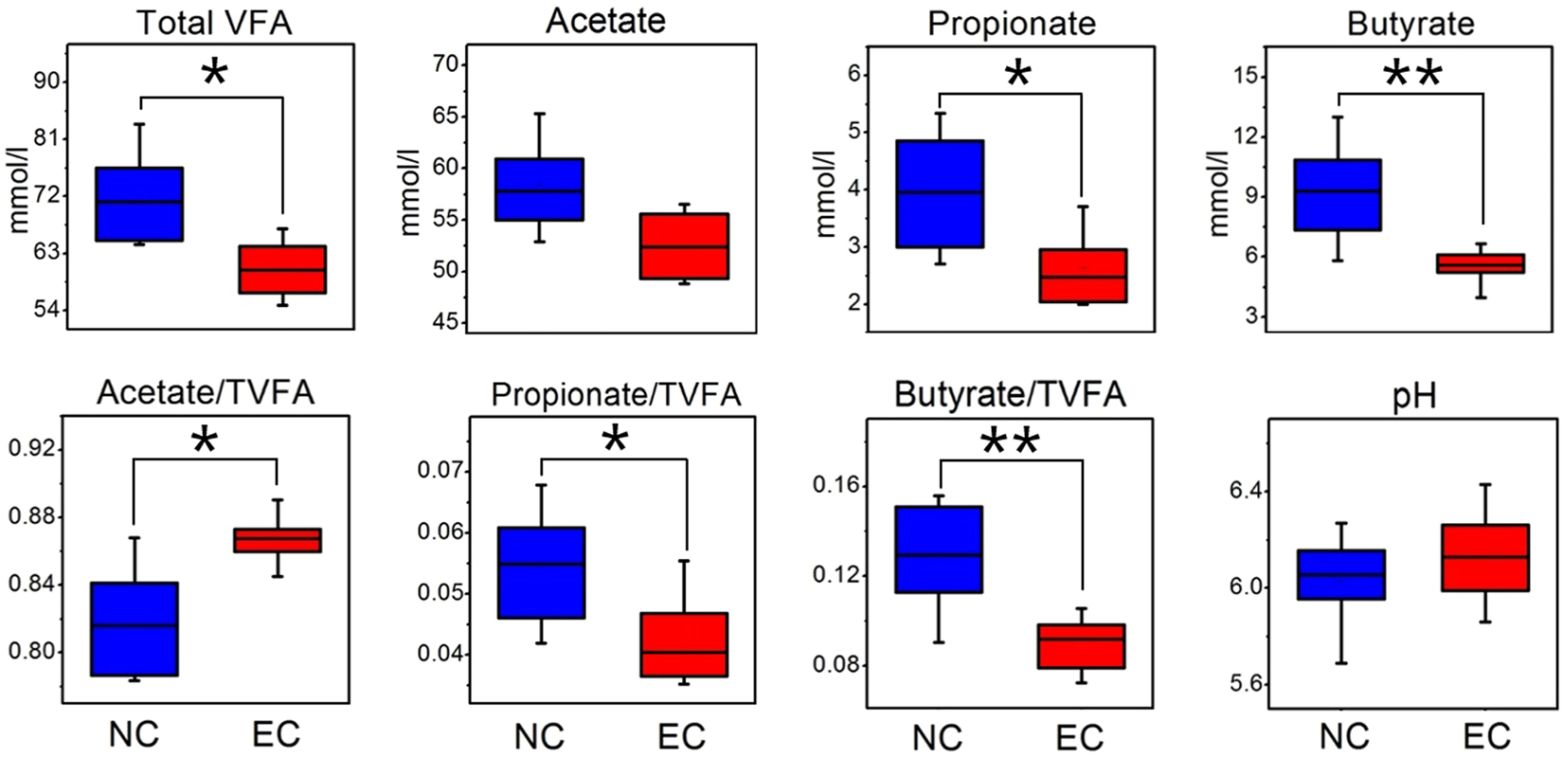
Effect of ERE on the concentration and molar proportions of the main VFAs and pH in caecal contents. Asterisks show significant differences between groups (*P < 0.05, **P < 0.01, Mann-Whitney U test).

The caecal pH and volatile fatty acid (VFA) concentrations of normal and ERE rabbits are presented in Fig. 9. The concentration (*P* < 0.05) and the molar proportion (*P* < 0.05) of propionate were significantly decreased in the caecum of ERE rabbits. In addition, the concentration (*P* < 0.01) and the molar proportions (*P* < 0.01) of butyrate were significantly decreased in EC compared with NC. All of this led to a lower concentration of total VFAs in the caecum of ERE rabbits (*P* < 0.05). The concentration of acetate also tended to be reduced (*P* = 0.06), but its molar proportion was significantly increased in ERE rabbits (*P* < 0.05).

### Relationship between caecal bacteria and fermentation parameters

**Figure 10:**
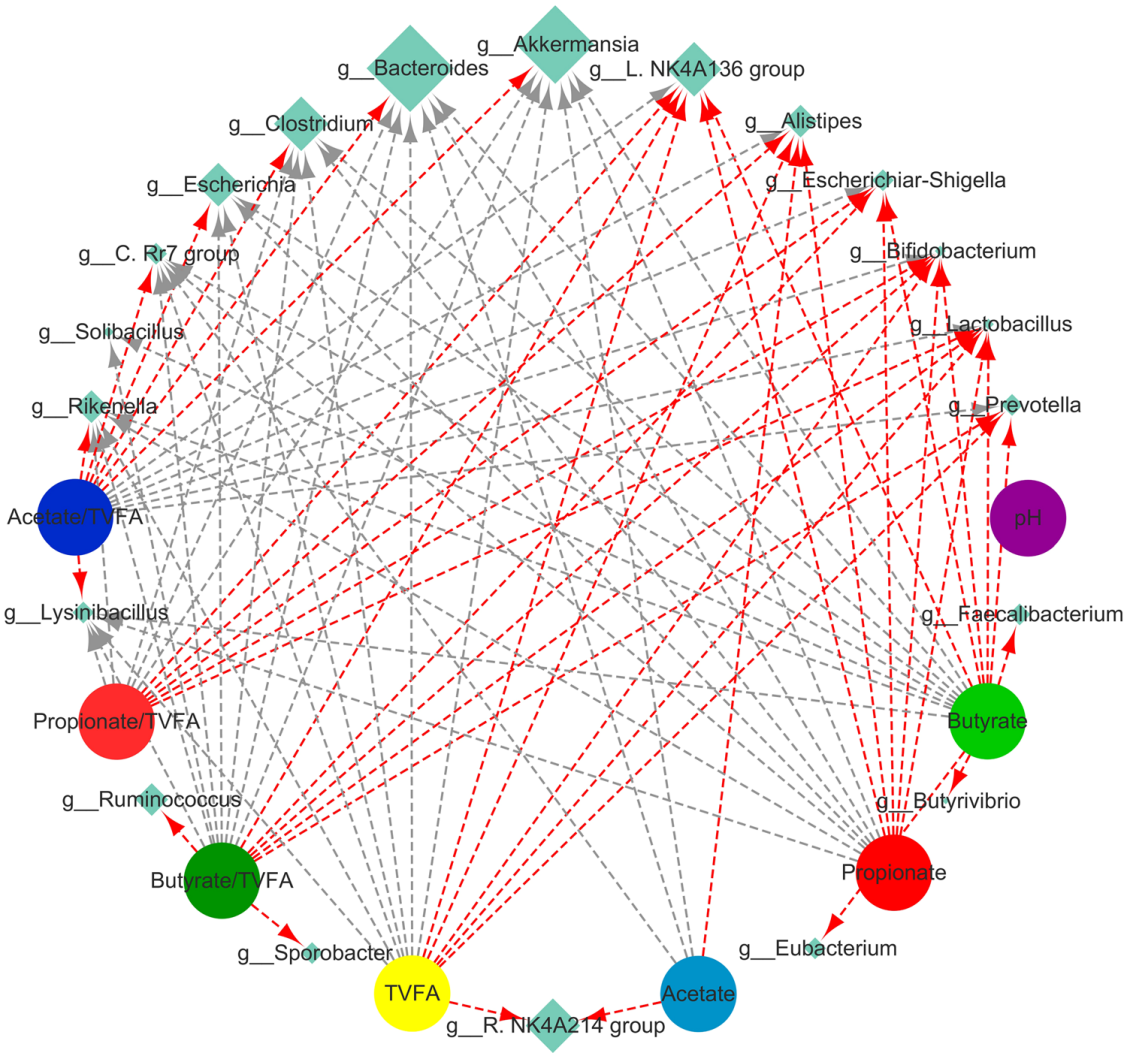
Correlation analysis between the relative abundances of caecal bacterial genera and caecal fermentation parameters. The network is based on the coefficients from Pearson’s correlation analysis with an absolute association score greater than 0.60 (*P* < 0.05). The sizes of the diamonds indicate the mean average relative abundance of caecal bacteria genera. The red lines indicate a positive correlation and the grey lines indicates a negative correlation between caecal bacterial genera and caecal fermentation parameters.

Pearson’s correlation was used to test the relationships between caecal bacteria and caecum fermentation parameters (Fig. 10). The relative abundance of caecal bacteria at the genus level was considered to be correlated with the pH or contents of the main VFAs if the correlation coefficients (r) were above 0.60 and *P* < 0.05. The concentration of TVFA was positively correlated with the abundances of the genera *Lachnospiraceae* NK4A136 group (r = 0.643, *P* < 0.05), *Lactobacillus* (r = 0.671, *P* < 0.05), *Bifidobacterium* (r = 0.634, *P* < 0.05), *Prevotella* (r = 0.602, *P* < 0.05), *Ruminococcaceae* NK4A214 group (r = 0.625, *P* < 0.05) and *Alistipes* (r = 0.651, *P* < 0.05), but negatively correlated with the abundances of genera *Rikenella* (r = −0.639, *P* < 0.05), *Bacteroides* (r = −0.679, *P* < 0.05), *Akkermansia* (r = −0.721, *P* < 0.01), *Clostridium*(r = −0.652, *P* < 0.05), *Lysinibacillus* (r = −0.628, *P* < 0.05), *Escherichia* (r = −0.639, *P* < 0.05) and *Christensenellaceae* R-7 group (r = −0.695, *P* < 0.05). The acetate concentration was positively correlated with the abundances of the genera *Alistipes* (r = 0.618, *P* < 0.05) *and Ruminococcaceae* NK4A214 group (r = 0.610, *P* < 0.05), negatively correlated with the abundance of the genera *Bacteroides* (r = −0.605, *P* < 0.05) and *Akkermansia* (r = −0.645, *P* < 0.05). The propionate concentration and molar proportion were positively correlated with the abundances of the genera *Escherichia-Shigella* (r = 0.676, *P* < 0.05; r = 0.721, *P* < 0.01), *Lachnospiraceae* NK4A136 group (r = 0.711, *P* < 0.01; r = 0.731, *P* < 0.01), *Lactobacillus* (r = 0.665, *P* < 0.05; r = 0.650, *P* < 0.05), *Bifidobacterium* (r = 0.641, *P* < 0.05; r = 0.633, *P* < 0.05) and *Alistipes* (r = 0.662, *P* < 0.05; r = 0.654, *P* < 0.05). The butyrate concentration and molar proportion were positively correlated with the abundances of the genera *Escherichia-Shigella* (r = 0.672, *P* < 0.01; r = 0.706, *P* < 0.01), *Lachnospiraceae* NK4A136 group (r = 0.710, *P* < 0.01; r = 0.721, *P* < 0.01), *Lactobacillus* (r = 0.699, *P* < 0.05; r = 0.685, *P* < 0.05), *Bifidobacterium* (r = 0.679, *P* < 0.01; r = 0.680, *P* < 0.05), *Prevotella* (r = 0.672, *P* < 0.05; r = 0.672, *P* < 0.05) and *Ruminococcus*(r = 0.601, *P* < 0.05; r = 0.620, *P* < 0.05). In contrast, the pH of the caecal contents was not significantly correlated with the abundance of any caecal bacterial genera (Fig. 10).

## Discussion

ERE is a severe digestive disease that mainly appears in post-weaning rabbits and has caused substantial economic losses in rabbit farming regions worldwide^1,17^. Although previous study had confirmed that ERE could be a bacterial disease and reported several suspect pathogens, the pathogenic bacteria of this disease still remained elusive^6,16^. In this study, rabbits were fed a low-fiber to establish an ERE model, and high-throughput sequencing was applied to examine and facilitate a comparison of the differences in microbial diversity in different parts of the gastrointestinal tract (stomach, small intestine and caecum) of normal and ERE rabbits. The α-diversity and qPCR data showed that the caecal samples had higher species richness and a more abundant bacterial population than the stomach and small intestine samples in normal rabbits, which was in line with previous studies^18^. Compared to the low pH of the stomach and aggressive intestinal fluids (e.g., bile, pancreatic juices) and the short transit time in the intestine, the environment in the caecum are more suitable for the propagation of bacteria^19,20^. In the current study, we found that the dominant bacterial phyla in the three parts of the gastrointestinal tract of normal rabbits were inhabited by Firmicutes, Proteobacteria, Bacteroidetes, Verrucomicrobia and Actinobacteria; however, the stomach and small intestine contents had higher relative abundances of Bacteroidetes and Actinobacteria and lower abundances of Firmicutes and Verrucomicrobia compared with the caecum. This phenomenon was similar to previous findings in other rodents: Bacteroidetes showed higher a relative abundance in the foregut (stomach and small intestine), and Firmicutes had a higher relative in the hindgut (caecum and colon)^18^. Earlier reports suggested that the main bacteria in the caecum were in the phyla Firmicutes, Bacteroidetes and Verrucomicrobia^21^. The results of the current study showed that the dominant bacterial phyla in the caecum of normal rabbits were Firmicutes (72.12% of the total sequence reads), Bacteroidetes (13.23%), Verrucomicrobia (3.96%) and Protrobacteria (5.09%), and this was quantitatively similar to a recent study conducted by Bäuerl *et al*.^16^.

We comparatively analysed the bacterial communities in the stomach, small intestine and caecum of normal and ERE rabbits. As shown in Figs 2, 5 and 6, the most important findings in the gastrointestinal microbiota of ERE rabbits are the remarkable dysbiosis, reduced taxonomic diversity and changes in community composition. The remarkable increase in Protobacteria, Bacteroidetes and Verrucomicrobia abundances in the caecum is most likely due to the increase in the genera *Escherichia*, *Bacteroides*, *Akkermansia* and *Clostridium*. This phenomenon was also observed in the stomach and small intestine in the present study. *Escherichia coli* appears frequently in ERE samples, regardless of whether the disease is produced under field conditions or in artificial induction^7,22^. Although enterohemorrhagic *E*. *coli* has been reported to cause colitis and haemolytic uremic syndrome in infant rabbits^23^, compelling evidence has indicated that enterohemorrhagic *E*. *coli* is not an aetiological agent of ERE, because ERE is fully reproduced by inoculation with TEC3 inoculum which is totally free of *E*. *coli*^5^. In this study, the abundance of *E*. *coli* remarkably increased from 6.05 (log_10_^copy number/ g content^) to 8.18 in the caecum, and this change was also present in the stomach and small intestine. High counts of opportunistic pathogens such as *E*. *coli* and also *Lysinibacillus* species were found in ERE rabbits but were rare or absent in normal rabbits; thus, these species may not be the cause of the disease but might well reflect the intestinal disorders and produce detrimental effects on the health of rabbits. Intestinal transit stopped after rabbit infection with ERE and led to abdominal distension and filling with large amounts of gas and fluid of liquid and caecal impaction, which could also explain the increased opportunistic pathogens (*E*. *coli*, and *C*. *perfringens*) under field conditions^4^.

*Clostridium spiroforme* and *Clostridium perfringens* are two common intestinal pathogenic bacteria that produce a similar lethal enterotoxin^24^ and lead to enterotoxaemia in rabbits^25^. High counts of *C*. *spiroforme* (6.50, log_10_^copy number/ g content^) and *C*. *perfringens* (7.28) were detected in the caeca of ERE rabbits. Previous reports showed that the counts of *C*. *perfringens* in caecal and ileal contents of rabbits affected by ERE were above 1.9 × 10^6^ and 1.58 × 10^5^ cfu/g^26^.

The genera *Akkermansia* have been proven to be important components of the caecal contents of healthy rabbits^27^, and are also present in the digestive tract of ERE rabbits. *Akkermansia muciniphila* has been suggested to be a mucin degrader and is regarded as a biomarker of healthy intestines in humans^28^. These species were highly enriched in ERE rabbits and might act as scavengers to clean the mucin caused by ERE^29^. In addition, *A*. *muciniphila* was reported to be an opportunistic pathogenic bacterium that exacerbates gut inflammation by facilitating microbial translocation to contact the intestinal epithelium^30^, as opportunistic pathogen bacteria may promote the development of this disease.

*Bacteroides* group, are one of the most prominent groups of rabbit intestinal microbiota^31^. As indigenous inhabitants, they play a crucial role in maintaining normal intestinal physiology and function. A more recent study indicated that the overgrowth of uncultured species in the genus *Bacteroides* was a potential causative agents of ERE^16^; however, the variations of the specific species in genus *Bacteroides* were not examined in that study. In the current study, we quantitatively analysed *Bacteroides* spp. in the digestive content of ERE rabbits, including *B*. *fragilis*, *B*. *ovatus*, *B*. *vulgatus*, *B*. *stercoris*, *B*. *thetaiotaomicron*, *B*. *caccae*, *B*. *eggerthii* and *B*. *uniformis*. As expected, all were detected and presented a significant increase after the rabbits became ill. *B*. *thetaiotaomicron* and *B*. *ovatus* carry out beneficial activities for the host through the degradation of polysaccharides^32^ and cause serum antibody responses in inflammatory bowel disease^33^. The other *Bacteroides* spp. were shown to exert different degrees of pathogenicity to the host, especially *B*. *fragilis*, which is an important opportunistic pathogen that produces an enterotoxin and causes acute diarrhoea^34^. Although direct evidence is lacking to conclude that these bacterial species are the causative agents of ERE, we can deduce that disordered gastrointestinal microbes may have a relationship with the onset of ERE to some extent. In future studies, more *Bacteroides* species should be isolated and transplanted into rabbits to examine their infectivity.

Another important founding in the ERE group was the increase in the abundance of the genera *Sphingomonas* in the stomach and *Enterobacter* in the stomach and small intestine. These bacteria were first detected in digestive samples from ERE rabbits. *Sphingomonas paucimobilis* is an opportunistic pathogen that is often infective to people with immunocompromised systems and causes meningitis^35^, pneumonia^36^ and diarrhoeal disease^37^. *Enterobacter sakazakii* is a rare pathogen and is known to cause severe neonatal septicaemia and meningitis^38^. Furthermore, many opportunistic pathogens were also observed at high abundances in one part of the digestive tract but were absent in others; for example, *Solibacillus* and *Anaerosporobacter* were remarkably increased in the small intestine, and *Rikenella* and *Cloacibacillus* were remarkably increased in the caecum of ERE rabbits compared with normal rabbits. To date, there are no reports showing that these bacteria are associated with ERE. However, we can speculate that these bacteria play a role in the development of ERE. Moreover, the results of the LEfSe analysis indicated that the stomach and small intestine contained more specific bacterial taxa than the caecum in ERE rabbits, suggesting that the pathogens and the emergence of ERE do not just occur in the caecum but also in the stomach and small intestine.

VFAs are the fermentation end products of microbes in the caecum and are an important tool to qualitatively evaluate microbial activity^39^. High concentrations of VFAs can inhibit the growth of bacteria of the family *Enterobacteriaceae*, especially *E*. *coli*, and provide energy for the body^40^. The present results showed that the ERE rabbits had lower TVFA concentrations and higher pH values, which might be due to the decreased abundances of *Ruminococcus*, *Alistipes*, *Faecalibacterium*, and other genera belonging to the family *Ruminococcaceae* (*R*. NK4A214 group and *R*. V9D2013 group) and *Lachnospiraceae* (*L*. NK4A136 group and *Lachnoclostridium*) in the caecum, these bacteria are the most abundant genera found in the rabbit gut^41^ and are capable of degrading plant polysaccharides to produce VFAs^42–44^. Moreover, butyric acid has been reported to play a critical role in maintaining intestinal health by providing energy for the colonic epithelium, acting as an inflammation and immune function modulator and inhibiting pathogens^45,46^. Since the abundances of the major butyric acid-producing bacteria *Butyrivibrio fibrisolvens*, *Faecalibacterium prausnitzii* and *Eubacterium* group were reduced, the concentration of butyric acid and its molar proportion were significantly decreased in ERE rabbits^47^. The remarkable decrease in TVFA, especially butyric acid may not be the origin of ERE but a consequence. Insufficient energy to maintain the normal physiological function of the intestine would result in weakened intestinal peristalsis and an overgrowth of pathogenic bacteria. Even if these speculations are accurate, they are indirect contributors to the development of ERE.

In summary, in the present study, the ERE model was established by feeding rabbits a low fibre diet; then, the microbes in the stomach, small intestine and caecum of normal and ERE rabbits were comparatively examined. The results showed great differences in the composition of the microbial in the three parts of the digestive tract in normal rabbits. The caecal contents had higher phylogenetic richness and bacterial abundance than the stomach and small intestine contents. The stomach and small intestine contents had higher abundances of Bacteroidetes and Actinobacteria, and the caecal contents had higher abundances of Firmicutes and Verrucomicrobia. Our results also suggest that ERE is a multifactorial disease, as stated before^7^, and ERE is mostly caused by variations in the composition of the microbial community in the stomach, small intestine and caecum or even the whole gastrointestinal tract. In addition to the widely accepted *Clostridium perfringens* and *Clostridium spiroforme*, the genera *Bacteroides fragilis*, *Akkermansia muciniphila* and *Enterobacter sakazakii* may also contribute to the onset of ERE. Furthermore, the decrease in the TVFA and butyric acid concentrations in the caecum of ERE rabbits was attributed to the reduced abundance of fibrolytic bacteria in the families *Ruminococcaceae* and *Lachnospiraceae*.

## Materials and Methods

The experimental protocol used in the present study was approved by the Animal Policy and Welfare Committee of the Agricultural Research Organization of Sichuan Province, China, and was in accordance with the guidelines of the Animal Care and Ethical Committee of Sichuan Agricultural University.

### Animals and experimental design

Sixty 5-week-old New Zealand specific pathogen-free rabbits of both genders (purchased from a rabbit farm in Tianquan, Ya’an, Sichuan) were used (mean weight of 890 ± 32.2 g). They were randomly assigned to two groups according to their weight (ERE group and normal group). All rabbits were placed in individual cages under controlled environmental conditions and were provided access to food and water *ad libitum* at Sichuan Agricultural University, China. To increase the incidence of ERE, the ERE group was fed a diet with a low fibre content according to a previous report^9^, and both diets lacked coccidiostats and antibiotic (CF = 9.49% vs.13.59%, ADF = 13.27% vs. 17.98%, NDF = 21% vs. 30%). The two diets were formulated according to the nutrient recommendations of de Blas and Wiseman^48^, and had similar energy and protein values, and the constituents and nutritional values are shown in Table 2. After seven days of adaptation and at the age of 55 days, six rabbits suffering typical ERE symptoms, such as anorexia, lethargy, abdominal distension, a hunched posture, caecal impaction and a watery sound in the gut, were selected at randomly from different cages of the low-fibre group. Additionally, six normal rabbits were selected randomly from the high-fiber group. All selected rabbits from each group were slaughtered and their stomach, intestinal and caecal contents were collected on the same day at 0800. The samples were frozen immediately at −80 °C until use.

**Table 2:** Ingredient and nutrient composition of the experimental diets.

Items	ERE	Normal		ERE	Normal
Ingredient (%)			Nutrient composition^b^		
Corn	13.05	7.78	DE (MJ/kg)	10.88	10.88
Broken rice	30.28	19.5	DM (%)	88.86	89.15
Soybean oil	0.52	3.38	CP (%)	17.00	17.00
Soybean meal	9.85	9.46	CF (%)	9.49	13.59
Cottonseed meal	12.17	11.06	NDF (%)	21.00	30.00
Wheat bran	6.37	10.11	ADF (%)	13.27	17.98
Clover meal	5.25	8.32	NFE (%)	49.7	43.52
Tea leaves	2.66	4.22	Ca (%)	0.85	0.85
Chinese wildrye	5.65	10.02	P (%)	0.76	0.76
Straw	6.78	10.82	AP (%)	0.40	0.40
Limestone powder	0.55	0.26	Met + Cys (%)	0.75	0.75
Calcium hydrogen phosphate	1.50	1.57	Lys (%)	0.83	0.83
HCL-Lys	0.25	0.25			
DL-Met	0.30	0.34			
Choline chloride	0.12	0.12			
Bentonite	3.20	1.20			
NaCl	0.50	0.50			
Premix compound^a^	1.00	1.00			
Total	100	100			

^a^Premix composition (by kg diet): vitamin A, 10000 IU; VD3, 1000 IU; VE, 40 mg; VK, 1 mg; VB6, 30 mg; nicotinic acid, 150 mg; Cu, 20 mg; Fe, 50 mg; Mn, 30 mg; Mg, 150 mg; Zn, 50 mg; I, 0.5 mg; Se, 0.05 mg; Co, 0.2 mg.

^b^DM, NDF, ADF, CF and NFE were determined values, and DE, CP, Ca, P, Lys and Met + Cys were calculated values.

### DNA extraction and 16s rRNA gene sequencing

Total genomic DNA from the stomach, intestinal and caecal contents was extracted using a stool DNA kit (Omega Bio-Tek, Norcross, GA, USA) according to the manufacturer’s instructions. The DNA concentration and quality were checked using a NanoDrop^TM^ 2000 (Thermo Scientific, Waltham, MA, USA) spectrophotometer. The 16S rRNA genes were amplified using primers 515 F (5′-GTGCCAGCMGCCGCGGTAA-3′) and 806 R (5′-GGACTACHVGGGTWTCTAAT-3′)^49^ for the V4 region of bacterial 16 S rRNA genes. The PCR mixture (35 μL) contained 1 × PCR buffer, 1.5 mM MgCl_2_, each deoxynucleoside triphosphate at 0.4 μM, each primer at 1.0 μM, 0.5 U of Ex Taq (TaKaRa, Dalian, China) and 10 ng of template DNA. The PCR amplification programme consisted of initial denaturation at 94 °C for 1 min, followed by 30 cycles (denaturation at 94 °C for 20 s, annealing at 30 °C for 30 s, and elongation at 72 °C for 45 s), and a final extension at 72 °C for 5 min. Three replicates of the PCR reactions for each sample were combined together. The PCR products were detected using 2% agarose gels, and samples with a bright main strip between 200–400 bp were chosen. Then, the PCR products were purified using the Omega Gel Extraction Kit (Omega Bio-Tek, Norcross, GA, USA). Sequencing libraries were generated using the TruSeq DNA PCR-Free Sample Preparation Kit (Illumina, San Diego, CA, USA) following the manufacturer’s recommendations and index codes were added. The library quality was assessed on a Qubit@ 2.0 fluorometer (Thermo Scientific, Waltham, MA, USA) and an Agilent Bioanalyzer 2100 system. Finally, the library was submitted to paired-end sequencing (2 × 250 bp) on the Illumina MiSeq platform (Rhonin Biotechnology Ltd, Chengdu, China).

### Quantitative real-time PCR (qPCR) analysis

qPCR was performed using the ABI-Prism 7900 Sequence Detection System (Applied Biosystems, Waltham, MA, USA) using optical grade 384-well plates. Sequences of the primers used for qPCR, which are specific for specific bacterial groups, are shown in Table S3. These oligonucleotide primers were synthesized commercially by Invitrogen (Shanghai, China). Triplicate samples were routinely used for the determination of DNA by qPCR, and the mean values were calculated. Each reaction (5 μl) composed of consisted Power SYBR PCR Master Mix (Applied Biosystems, Waltham, MA, USA), 1 μl of each of the specific primers at a concentration of 100 nM, and 1 μl of template DNA. The PCR reaction conditions for DNA amplification consisted of one cycle of pre-denaturation at 95 °C for 1 min; 40 cycles of denaturation at 95 °C for 20 s, annealing at 50–63 °C for 20 s (Table S3), and 72 °C for 50 s and finally one cycle of 94 °C for 15 s. The melting curve analysis was made after amplification to assess the specificity of the amplification reaction. The melting curves were obtained by slow heating at temperatures from 60 to 95 °C at a rate of 0.2 °C/s, with continuous fluorescence collection.

Specific standard curves for each bacterium were generated to quantify the copy numbers in test samples by constructing standard plasmids, as described previously by Chen *et al*.^50^. The specific PCR amplification product of each bacteria was recycled and purified using the AxyPrep NDA Gel Extraction Kit (Axygen, Santa Clara, CA, USA), cloned into a pGEM-T Easy Vector, introduced into *E. coli*. JM109, and finally inoculated in 1 ml of Luria-Bertani medium at 37 °C for 24 h. The recombinant plasmid of each bacteria was isolated and purified using an Omega plasmid mini kit, and standard plasmids of all bacteria were constructed successfully. The DNA concentrations of the standard plasmids were measured using a NanoDropTM 2000 (Thermo Scientific, Waltham, MA, USA) spectrophotometer. The copy numbers were calculated using the following formula: (6.0233 × 1023 copies/mol × DNA concentration (μg/μl))/(660 × 106 × DNA length (bp)). A specific standard curve was prepared using serial 10-fold dilutions of plasmid DNA with a bacterial population ranging from 2 to 10 log10 copy numbers/μl. Each standard curve was constructed by a linear regression of the plotted points, and cycle threshold (CT) values were plotted against the log10 copy numbers. The bacterial concentration in each sample was calculated by interpolation of the obtained Ct values from the standard curves.

### Quantification of volatile fatty acids

The VFA concentrations in caecal content samples were analysed by gas chromatography following the procedure described by Chen *et al*.^50^. One gram of each content sample was thawed and resuspended in 2 ml of distilled water in a screw-capped tube. The samples were vortex-mixed and centrifuged (12,000 × g) at 4 °C for 10 min. The supernatants (1 ml volume each) were transferred into Eppendorf tubes and mixed with 0.2 ml of metaphosphoric acid. The tubes were placed at 4 °C for 30 min and then centrifuged (12,000 × g, 4 °C, 10 min). Then, 1 μL of each supernatant was taken for analysis of VFAs by gas chromatography (Varian CP-3800, Agilent Technologies, Palo Alto, CA, USA). A flame ionization detector was used with a column temperature between 100 °C and 150 °C and a detector temperature of 250 °C to determine the acetate, propionate, and butyrate concentrations. A polyethylene glycol column was operated with highly purified N2 at 1.8 ml/min as the carrier gas. The detection limit for VFAs was 0.1 mmol/L.

### Statistical and bioinformatics analysis

Paired-end reads from the original NDA fragments were theoretically merged using FLASH (V1.2.7, http://ccb.jhu.edu/software/FLASH/). Paired-end reads were assigned to each sample according to their unique barcode. Sequences of high quality (length > 200 bp, without the ambiguous base ‘N’, and with an average base quality score >30) were screened for chimeras using the UCHIME algorithm. Sequences were clustered into OTUs at the 97% identity threshold using the UPARSE-OTU ref algorithm. Representative sequences were selected for each OTU. Taxonomy was assigned using the Greengenes database (http://greengenes.lbl.gov/cgi-bin/nph-index.cgi), and the representative sequences were aligned using PyNAST. The analysis of alpha diversity, which included calculation of the observed species and the Chao1, ACE, Shannon, and Simpson indices, was conducted with Vegan (version 2.0–2.R CRAN package). Rarefaction curves were generated based on these three metrics. To eliminate the influence of sequence depth on community diversity, the OTU table was rarified to make all samples contain the same sequence number of 7762. Principal component analysis (PCA) was applied to reduce the dimensions of the original community data. A heatmap was generated using the heatmap function of R (http://www.r-project.org/) and family information for the two groups. The linear discriminant analysis (LDA) effect size (LEfSe) method was performed to identify the bacterial taxa differentially represented between groups at the genus or higher taxonomic levels, which would help reveal biomarkers^51^. All statistical analyses were run using SAS v9.4 (SAS Institute Inc., Cary, NC, USA). Differences between means were considered to be significant at the *P* < 0.05 level.

## Electronic supplementary material

Supplementary Figure 1

Supplementary Table 1

Supplementary Table 2

Supplementary Table 3

3D PCOA Plot original data

alpha diversity original data

cecal ph and vfa original data

correlation analysis original data

NC-EC lefse analysis original data

NI-EI lefse analysis original data

ES-EI-EC lefse analysis original data

NS-ES lefse analysis original data

NS-NI-NC lefse analysis original data

OTU table original data

taxa summary original data
