# Supplementary material for: The underlying microbial mechanism of epizootic rabbit enteropathy triggered by a low fiber diet
Source: Sci Rep. 2018 Aug 21;8:12489. doi: 10.1038/s41598-018-30178-2 (PMC6104036; doi:10.1038/s41598-018-30178-2)
Supplement: Supplementary file 1 — Supplementary Figure 1 [file 41598_2018_30178_MOESM1_ESM.pdf]

The underlying microbial mechanism of epizootic rabbit enteropathy triggered by a low fiber diet

DingXing Jin[a*], HuaWei Zou[a*], SiQiang Liu[a], LiZhi Wang[a], Bai Xue[a], De Wu[a], Gang Tian[a], Jingyi Cai[a], TianHai Yan[b], ZhiSheng Wang[a] & QuanHui Peng[a]

[a] Institute of Animal Nutrition, Key Laboratory of Bovine Low-Carbon Farming and Safe Production, Sichuan Agricultural University, Ya'an, Sichuan, 625014, PR China

[b] Agri-Food and Biosciences Institute, Hillsborough, Co. Down BT26 6DR, United Kingdom

*This two authors contributed equally to this paper.

Correspondence and requests for materials should be addressed to Q.P. and Z.W. (email: pengquanhui@126.com , wangzs67@163.com)

Address: Animal nutrition institute, Xinkang road 46#, Yucheng, Ya'an, Sichuan province. Post code: 625014

Tel: +86 15283511570

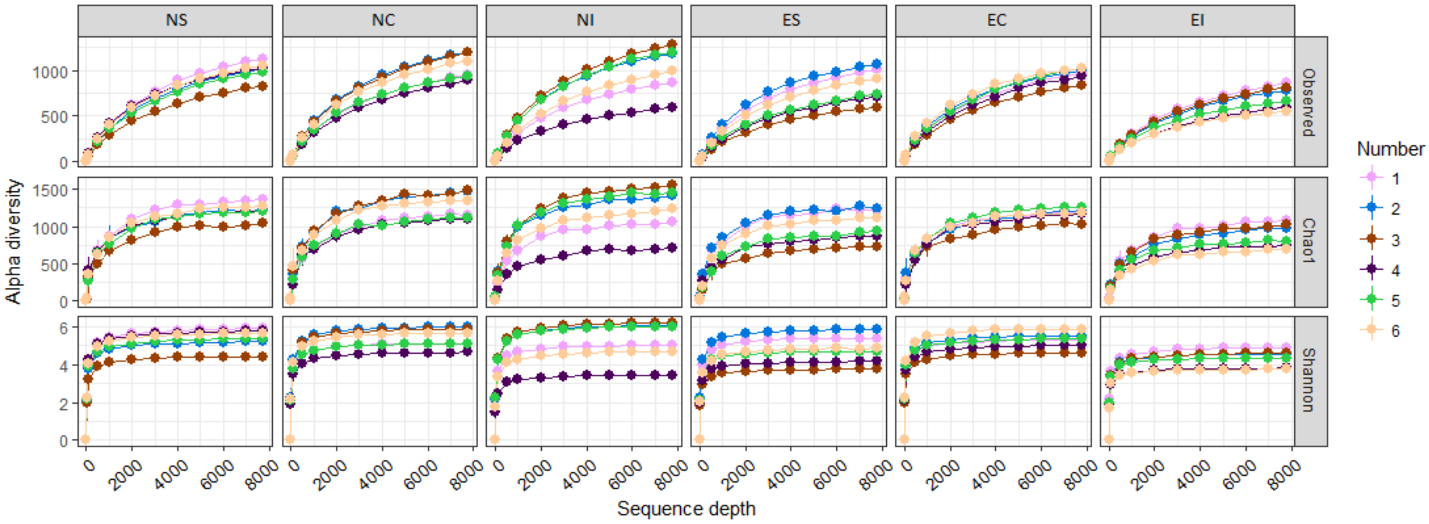

Figure S1. Diversity estimation of the 16S rRNA gene libraries in the stomach, small intestine and caecum of normal and ERE rabbits from the sequencing data.
