# Supplementary material for: The underlying microbial mechanism of epizootic rabbit enteropathy triggered by a low fiber diet
Source: Sci Rep. 2018 Aug 21;8:12489. doi: 10.1038/s41598-018-30178-2 (PMC6104036; doi:10.1038/s41598-018-30178-2)
Supplement: Supplementary file 2 — Supplementary Table 1 [file 41598_2018_30178_MOESM2_ESM.pdf]

The underlying microbial mechanism of epizootic rabbit enteropathy triggered by a low fiber diet

DingXing Jin[a*], HuaWei Zou[a*], SiQiang Liu[a], LiZhi Wang[a], Bai Xue[a], De Wu[a], Gang Tian[a], Jingyi Cai[a], TianHai Yan[b], ZhiSheng Wang[a] & QuanHui Peng[a]

[a] Institute of Animal Nutrition, Key Laboratory of Bovine Low-Carbon Farming and Safe Production, Sichuan Agricultural University, Ya'an, Sichuan, 625014, PR China

[b] Agri-Food and Biosciences Institute, Hillsborough, Co. Down BT26 6DR, United Kingdom

*This two authors contributed equally to this paper.

Correspondence and requests for materials should be addressed to Q.P. and Z.W. (email: pengquanhui@126.com , wangzs67@163.com)

Address: Animal nutrition institute, Xinkang road 46#, Yucheng, Ya’an, Sichuan province. Post code: 625014

Tel: +86 15283511570

Table S1. Relative abundance (%) of bacterial at different classification level in the three parts of the digestive tracts in normal and ERE rabbits. The relative abundance ≥ 0.05% was present in this table.

Item	S			I			C			*P*-value
	NS	ES	*P*	NI	EI	*P*	NC	EC	*P*	Normal
p__Firmicutes	44.60[b]	25.73	<0.01	41.69[b]	31.71	<0.01	72.12[a]	47.72	<0.01	<0.01
c__Clostridia	33.00[c]	17.43	<0.01	37.61[b]	22.98	<0.01	65.70[a]	39.78	<0.01	<0.01
o__Clostridiales	32.80[c]	17.42	<0.01	37.57[b]	23.01	<0.01	65.66[a]	39.72	<0.01	<0.01
f__Ruminococcaceae	16.84[c]	6.69	<0.01	24.72[b]	9.24	<0.01	32.77[a]	13.70	<0.01	<0.01
g__Ruminococcaceae NK4A214 group	3.10[b]	2.15	0.13	3.14[b]	0.53	<0.01	7.27[a]	4.55	<0.01	<0.01
g__Ruminococcaceae V9D2013 group	1.52[b]	1.09	0.35	4.61[a]	0.37	<0.01	5.20[a]	1.90	<0.01	<0.01
g__Ruminococcaceae UCG-014 group	0.05[b]	0.16	0.05	2.54[a]	0.52	<0.01	2.37[a]	2.13	0.64	<0.01
g__Ruminococcaceae UCG-013 group	0.15[b]	0.23	0.52	0.24[b]	0.15	0.43	1.01[a]	0.83	0.43	0.03
g__Ruminococcaceae UCG-005 group	0	0	-	0.24	0.05	0.04	0.22	0.11	0.46	0.11
g__Ruminococcus	1.85[b]	0.09	0.03	2.03[b]	0.56	<0.01	3.77[a]	0.89	<0.01	<0.01
g__Subdoligranulum	1.05[b]	0.65	0.24	1.13[b]	0.98	0.10	2.41[a]	1.85	0.23	<0.01
g__Ruminiclostridium	3.74[a]	1.43	<0.01	4.73[a]	0.84	<0.01	1.44[b]	1.29	0.46	0.02
g__Anaerotruncus	0.22[c]	0.14	0.14	0.39[b]	0.29	0.58	0.63[a]	0.31	<0.01	<0.01
g__Faecalibacterium	0.76	0.07	0.06	0.32	0	0.02	1.59	0.14	<0.01	0.08
g__Flavonifractor	0.54[a]	0	0.02	0.09[b]	0.12	0.55	0.69[a]	0.12	<0.01	<0.01
g__Papillibacter	0.60	0	0.02	0	0	-	0.91	0.13	<0.01	<0.01
f__Lachnospiraceae	5.91[b]	3.74	0.01	8.66[b]	5.78	0.07	23.66[a]	11.16	<0.01	<0.01

g__Lachnospiraceae NK4A136 group	2.30[c]	1.55	0.10	5.40[b]	2.54	0.02	8.46[a]	3.26	<0.01	<0.01
g__Lachnoclostridium	0.87[b]	0.63	0.45	1.40[a]	0.87	0.10	1.49[b]	1.09	0.13	0.01
g__Sporobacter	0.16[b]	0.21	0.57	0.22[b]	0.52	0.16	1.69[a]	1.48	0.51	<0.01
g__Fusicatenibacter	0.30[b]	0.21	0.35	0.34[b]	0.15	0.05	2.35[a]	0.61	<0.01	<0.01
g__Butyrivibrio	0.35	0	<0.01	0.87	0	<0.01	0.66	0.05	<0.01	0.57
g__Tyzzerella	0.51[a]	0.33	0.51	0.49[a]	0.13	0.06	0.18[b]	0.19	0.86	0.03
g__Anaerosporobacter	0.24[b]	0.21	0.60	0.35[a]	1.54	<0.01	0.42[a]	0.29	0.05	<0.01
g__Blautia	0.10	0.07	0.47	0.18	0.13	0.62	0.27	0.11	0.04	0.06
f__Eubacteriaceae	4.91[a]	0.67	<0.01	1.17[c]	0.24	0.06	4.64[b]	3.10	0.03	<0.01
g__Eubacterium	1.34[a]	0.45	0.04	0.76[c]	0.19	0.30	1.40[b]	1.10	0.49	<0.01
f__Clostridiaceae	2.06[b]	5.17	<0.01	2.46[b]	6.84	<0.01	3.92[a]	9.78	<0.01	0.03
g__Clostridium	0.89	3.83	<0.01	1.10	3.12	<0.01	1.27	6.52	<0.01	0.09
f__Christensenellaceae	0.40	0.29	0.33	0.41	0.26	0.39	0.64	1.58	<0.01	0.14
g__Christensenellaceae R-7 group	0.33	0.28	0.72	0.40	0.24	0.33	0.64	1.56	<0.01	0.10
f__Family XI	0.25	0.56	0.14	0.21	0.53	0.16	0.18	0.23	0.52	0.72
g__Sedimentibacter	0.25	0.56	0.14	0.21	0.53	0.16	0.18	0.23	0.52	0.72
o__Halanaerobiales	0	0	-	0	0	-	0	0.11	0.43	-
f__Halobacteroidaceae	0	0	-	0	0	-	0	0.11	0.43	-
c__Bacilli	3.87	6.45	0.02	2.26	7.03	<0.01	3.06	4.06	0.03	0.17
o__Bacillales	2.40	6.13	<0.01	1.75	6.93	<0.01	0.95	3.95	<0.01	0.06
f__Planococcaceae	0.65	5.47	<0.01	0.53	5.87	<0.01	0.26	3.43	<0.01	0.33
g__Lysinibacillus	0.29	3.16	<0.01	0.18	1.87	<0.01	0.11	2.51	<0.01	0.21
g__Solibacillus	0.32	0.25	0.62	0.30	2.22	<0.01	0.13	0.90	<0.01	0.45
f__Bacillaceae	1.09	0.55	0.05	0.90	0.81	0.77	0.53	0.47	0.78	0.14
g__Bacillus	1.09	0.55	0.05	0.90	0.81	0.77	0.53	0.47	0.78	0.14
o__Lactobacillales	2.44	0.22	0.06	1.89	0.08	0.11	2.44	0.08	<0.01	0.63
f__Lactobacillaceae	2.44	0.22	0.06	1.89	0.08	0.11	2.38	0.08	<0.01	0.63
g__Lactobacillus	2.24	0.09	0.04	1.89	0.05	0.01	1.08	0.08	<0.01	0.48
c__Erysipelotrichia	5.58[a]	0.21	<0.01	0.45[b]	0.64	0.37	0.27[b]	0.25	0.80	<0.01
o__Erysipelotrichales	4.24[a]	0.21	<0.01	1.66[b]	0.51	0.41	0.27[b]	0.25	0.80	<0.01
f__Erysipelotrichaceae	4.24[a]	0.21	<0.01	1.66[b]	0.51	0.41	0.27[b]	0.25	0.80	<0.01
g__Allobaculum	2.29[a]	0.11	<0.01	0.35[b]	0.15	0.25	0.11[b]	0.11	0.83	<0.01
g__Turicibacter	0.68	0.06	0.08	0.43	0.29	0.70	0.11	0.12	0.83	0.51
c__Negativicutes	2.14	1.50	0.22	1.28	1.54	0.64	2.95	3.59	0.58	0.16
o__Selenomonadales	2.14	1.46	0.19	1.28	1.37	0.44	2.78	3.27	0.69	0.25
f__Acidaminococcaceae	1.76	1.63	0.87	0.92	1.13	0.49	1.78	2.88	0.72	0.19
g__Phascolarctobacterium	1.76	0.95	0.07	0.91	1.13	0.50	0.27	1.37	0.03	0.19
f__Veillonellaceae	0.38	0.49	0.48	0.36	0.25	0.40	0.34	0.36	0.76	0.96
g__Megasphaera	0.19	0.38	0.13	0.25	0.21	0.65	0.25	0.27	0.83	0.77

p__Proteobacteria	27.50^{a}	52.25	<0.01	18.28^{b}	16.48	0.23	5.09^{c}	10.86	<0.01	<0.01
c__Gammaproteobacteria	14.03^{a}	22.47	<0.01	10.44^{b}	14.64	0.01	2.79^{c}	9.25	<0.01	<0.01
o__Enterobacteriales	3.67^{a}	17.64	<0.01	3.50^{a}	13.01	<0.01	1.34^{b}	7.19	<0.01	<0.01
f__Enterobacteriaceae	3.67^{a}	17.64	<0.01	3.50^{a}	13.01	<0.01	1.34^{b}	7.19	<0.01	<0.01
g__Escherichia-Shigella	1.85^{b}	1.73	0.77	2.78^{b}	1.18	<0.01	2.62^{a}	0.63	<0.01	0.03
g__Enterobacter	0.34^{a}	4.12	<0.01	0.13^{ab}	5.30	<0.01	0.07^{b}	0.12	0.49	0.02
g__Escherichia	0.74	3.80	<0.01	0.33	4.39	<0.01	0.11	6.39	<0.01	0.64
o__Pseudomonadales	7.08^{a}	4.01	0.03	4.36^{b}	1.13	<0.01	0.82^{c}	1.45	0.16	<0.01
f__Pseudomonadaceae	5.05^{a}	4.17	0.02	4.03^{b}	1.11	<0.01	0.82^{c}	1.34	0.19	<0.01
g__Pseudomonas	2.71^{a}	4.17	0.05	3.69^{b}	1.09	<0.01	0.66^{c}	1.21	0.17	<0.01
o__Xanthomonadales	1.53^{a}	0.34	<0.01	1.00^{a}	0.16	0.02	0.22^{b}	0.19	0.71	<0.01
f__Xanthomonadaceae	1.48^{a}	0.32	<0.01	0.97^{a}	0.15	0.02	0.18^{b}	0.18	0.94	<0.01
g__Xanthomonas	1.32^{a}	0.29	<0.01	0.80^{b}	0.15	0.02	0.18^{c}	0.18	0.95	<0.01
o__Oceanospirillales	1.39^{a}	0.12	<0.01	0.19^{b}	0	0.20	0.20^{b}	0	0.33	<0.01
f__Halomonadaceae	1.39^{a}	0.11	<0.01	0.19^{b}	0	0.20	0.20^{b}	0	0.34	<0.01
g__Halomonas	1.39^{a}	0.11	<0.01	0.19^{b}	0	0.20	0.20^{b}	0	0.34	<0.01
o__Acidithiobacillales	0.33	0.32	0.93	0.36	0.24	0.36	0.14	0.23	0.27	0.27
c__Alphaproteobacteria	7.45^{a}	24.44	<0.01	4.41^{b}	0.88	<0.01	0.14^{c}	0.10	0.18	<0.01
o__Sphingomonadales	3.86^{a}	15.55	<0.01	2.86^{a}	0.49	<0.01	0.66^{b}	0.54	0.40	<0.01
f__Sphingomonadaceae	3.85^{a}	12.81	<0.01	2.86^{a}	0.49	<0.01	0.63^{b}	0.52	0.43	<0.01
g__Sphingomonas	3.37^{a}	13.93	<0.01	2.12^{a}	0.41	<0.01	0.59^{b}	0.50	0.53	<0.01
o__Rhizobiales	2.41^{a}	6.56	<0.01	0.96^{b}	0.25	<0.01	0.42^{b}	0.29	0.27	<0.01
f__Methylobacteriaceae	1.35^{a}	2.32	0.13	0.50^{ab}	0.14	0.09	0.27^{b}	0.20	0.42	0.04
g__Methylobacterium	1.35^{a}	2.32	0.28	0.50^{ab}	0.14	0.09	0.27^{b}	0.20	0.42	0.04
f__Hyphomicrobiaceae	0.36	2.34	<0.01	0.19	0	0.07	0	0	-	0.02
o__Rhodospirillales	0.61	0.29	0.32	0.29	0.08	0.07	0.14	0.10	0.68	0.23
f__Rhodospirillaceae	0.31	0.26	0.79	0.24	0.08	0.10	0.11	0.10	0.72	0.53
g__Defluviicoccus	0.26	0.11	0.48	0.16	0.08	0.46	0.10	0.06	0.61	0.69
o__Caulobacterales	0.47	0.43	0.85	0.24	0.07	0.03	0.09	0.10	0.89	0.05
f__Caulobacteraceae	0.42^{a}	0.42	0.95	0.21^{ab}	0.05	0.03	0.09^{b}	0.09	0.75	0.04
g__Brevundimonas	0.24	0.28	0.77	0.18	0	0.04	0.08	0.09	0.68	0.31
c__Betaproteobacteria	4.79^{a}	4.82	0.99	2.67^{b}	0.47	<0.01	0.67^{c}	0.53	0.45	<0.01
o__Burkholderiales	3.85^{a}	4.29	0.73	2.34^{b}	0.41	<0.01	0.58^{c}	0.50	0.61	<0.01
f__Oxalobacteraceae	1.93^{a}	2.69	0.48	1.10^{ab}	0.15	<0.01	0.20^{b}	0.17	0.64	0.01
g__Janthinobacterium	1.16^{a}	2.31	0.15	0.83^{ab}	0.10	<0.01	0.11^{b}	0.11	0.98	<0.01
f__Comamonadaceae	0.44	0.55	0.68	0.35	0.07	0.03	0	0.07	0.21	0.05
g__Comamonas	0.44	0.55	0.68	0.35	0.07	0.03	0	0.07	0.21	0.05
f__Burkholderiaceae	0.80^{a}	0.60	0.37	0.36^{b}	0.13	0.09	0.25^{b}	0.18	0.46	<0.01
g__Burkholderia	0.78^{a}	0.57	0.31	0.35^{b}	0.12	0.08	0.24^{b}	0.18	0.49	<0.01
c__Deltaproteobacteria	1.38^{a}	0.88	0.18	0.71^{b}	0.48	0.35	0.22^{b}	0.16	0.51	<0.01

o__Desulfovibrionales	0.80	0.77	0.94	0.45	0.42	0.84	0.15	0.13	0.80	0.06
f__Desulfovibrionaceae	0.80	0.77	0.94	0.45	0.42	0.84	0.15	0.13	0.80	0.06
g__Bilophila	0.57	0.49	0.81	0.28	0.36	0.62	0.10	0.12	0.83	0.18
o__Desulfuromonadales	0.30	0.08	0.04	0.23	0	0.27	0.05	0	0.40	0.25
f__Desulfuromonadaceae	0.20	0.08	0.10	0.22	0	0.28	0	0	-	0.39
g__Desulfuromonas	0.20	0.08	0.10	0.22	0	0.28	0	0	-	0.39
p__Bacteroidetes	18.93^{b}	16.39	0.18	32.33^{a}	41.06	<0.01	13.23^{c}	24.20	<0.01	<0.01
c__Bacteroidia	13.34^{b}	15.93	0.25	26.64^{a}	40.89	<0.01	12.92^{b}	24.00	<0.01	<0.01
o__Bacteroidales	13.06^{b}	15.60	0.26	24.38^{a}	40.24	<0.01	12.77^{b}	23.79	<0.01	<0.01
f__Bacteroidaceae	4.08^{b}	10.42	<0.01	8.52^{a}	20.45	<0.01	2.60^{b}	14.88	<0.01	<0.01
g__Bacteroides	3.93^{b}	8.42	0.01	4.52^{a}	20.45	<0.01	2.60^{b}	17.09	<0.01	<0.01
f__Rikenellaceae	6.08^{b}	2.32	<0.01	12.36^{a}	10.77	0.03	7.65^{b}	5.20	0.03	<0.01
g__Alistipes	1.82^{b}	0.92	0.02	1.88^{b}	0.91	<0.01	3.84^{a}	0.75	<0.01	<0.01
g__Rikenella	0.57	1.38	0.55	0.81	1.10	0.50	0.36	4.11	0.01	0.41
g__dgA-11 gut group	0.22	0.08	0.10	0.51	1.23	<0.01	0.29	0.35	0.80	0.04
g__Rikenellaceae RC9 gut group	0.30	0	<0.01	0.47	0	<0.01	0.07	0	<0.01	<0.01
f__Prevotellaceae	0.89	0.07	<0.01	2.04	0.05	<0.01	1.95	0	<0.01	0.08
g__Prevotella	0.89	0.07	<0.01	1.87	0.05	<0.01	1.95	0	<0.01	0.10
f__Bacteroidales S24-7 group	2.15	1.76	0.63	2.44	5.94	<0.01	2.24	3.58	0.02	0.93
c__Sphingobacteriia	5.07^{a}	0.21	<0.01	5.17^{a}	0.14	<0.01	0.26^{b}	0.14	0.49	<0.01
o__Sphingobacteriales	5.01^{a}	0.19	<0.01	4.26^{b}	0.13	<0.01	0.24^{b}	0.14	0.51	<0.01
f__Sphingobacteriaceae	4.40^{a}	0.18	<0.01	3.89^{b}	0.13	<0.01	0.22^{b}	0.14	0.56	<0.01
g__Pedobacter	1.15^{a}	0.10	<0.01	0.96^{b}	0.06	<0.01	0.11^{b}	0.07	0.48	<0.01
g__Mucilaginibacter	0.37	0.08	0.18	0.30	0.06	0.15	0.10	0.07	0.69	0.43
p__Verrucomicrobia	0.52^{b}	3.22	0.19	0.74^{b}	9.42	<0.01	4.29^{a}	13.27	<0.01	<0.01
c__Verrucomicrobiae	0.44^{b}	3.13	0.23	0.73^{b}	9.35	<0.01	4.19^{a}	13.24	<0.01	<0.01
o__Verrucomicrobiales	0.44^{b}	3.13	0.23	0.73^{b}	9.21	<0.01	4.19^{a}	13.09	<0.01	<0.01
f__Verrucomicrobiaceae	0.44^{b}	3.13	0.23	0.73^{b}	9.21	<0.01	4.19^{a}	13.07	<0.01	<0.01
g__Akkermansia	0.42^{b}	3.08	0.04	0.73^{b}	9.21	<0.01	3.36^{a}	13.06	<0.01	<0.01
p__Actinobacteria	5.10	1.32	<0.01	4.05	0.53	0.01	2.89	1.01	<0.01	0.19
c__Actinobacteria	3.49	1.10	0.01	3.30	0.40	0.03	2.69	0.59	<0.01	0.70
o__Micrococcales	0.74	0.54	0.49	1.05	0.24	0.18	0.31	0.24	0.45	0.34
f__Dermabacteraceae	0.61	0.39	0.43	0.46	0.15	0.14	0.16	0.11	0.30	0.19
g__Brachybacterium	0.61	0.39	0.43	0.46	0.15	0.14	0.16	0.11	0.30	0.19
f__Micrococcaceae	0.12	0.12	0.96	0.12	0.07	0.36	0.06	0.09	0.26	0.27
g__Kocuria	0.11	0.12	0.82	0.08	0.07	0.86	0.05	0.08	0.27	0.19
o__Bifidobacteriales	0.95	0.13	<0.01	1.61	0.06	<0.01	1.48	0.07	<0.01	0.82

f__Bifidobacteriaceae	0.95	0.13	<0.01	1.61	0.06	<0.01	1.48	0.07	<0.01	0.82
g__Bifidobacterium	0.95	0.13	<0.01	1.61	0.06	<0.01	1.48	0.07	<0.01	0.82
c__Coriobacteriia	1.46[a]	0.11	0.02	0.51[b]	0.05	0.06	0.09[b]	0.32	0.20	0.02
o__Propionibacteriales	1.29[a]	0.11	0.02	0.50[ab]	0.05	0.06	0.09[b]	0.32	0.20	0.02
f__Propionibacteriaceae	1.29[a]	0.11	0.02	0.50[ab]	0.05	0.06	0.09[b]	0.32	0.20	0.02
g__Haloactinopolyspora	1.20[a]	0.10	0.03	0.49[ab]	0.05	0.09	0.08[b]	0.30	0.18	0.04
c__Acidimicrobiia	0.30	0.10	<0.01	0.23	0.08	0.09	0.13	0.10	0.38	0.07
o__Acidimicrobiales	0.30	0.10	<0.01	0.23	0.08	0.09	0.12	0.10	0.55	0.06
f__uncultured	0.16	0.07	0.07	0.11	0.08	0.33	0.08	0.06	0.52	0.14
g__uncultured	0.16	0.07	0.07	0.11	0.08	0.33	0.08	0.06	0.52	0.14
p__Chloroflexi	1.16[a]	0.79	0.24	1.01[a]	0.35	0.10	0.49[b]	0.64	0.12	<0.01
c__Anaerolineae	0.87[a]	0.69	0.54	0.89[a]	0.25	0.14	0.32[b]	0.46	0.13	0.02
o__Anaerolineales	0.51	0.63	0.67	0.58	0.23	0.44	0.28	0.41	0.11	0.23
f__Anaerolineaceae	0.51	0.63	0.67	0.58	0.23	0.44	0.28	0.41	0.11	0.23
g__uncultured	0.41	0.57	0.57	0.36	0.16	0.04	0.21	0.31	0.13	0.31
c__Gitt-GS-136	0.25	0.08	0.17	0.09	0.09	0.90	0.10	0.16	0.13	0.18
o__uncultured	0.25	0.08	0.17	0.08	0.07	0.76	0.09	0.12	0.31	0.20
p__Synergistetes	0.29	0.44	0.35	0.16	0.22	0.60	0.52	1.89	0.08	0.49
c__Synergistia	0.29	0.44	0.35	0.16	0.22	0.60	0.52	1.89	0.08	0.49
o__Synergistales	0.29	0.44	0.35	0.16	0.22	0.60	0.52	1.89	0.08	0.49
f__Synergistaceae	0.29	0.44	0.35	0.16	0.22	0.60	0.52	1.89	0.08	0.49
g__Cloacibacillus	0.19	0.21	0.86	0.12	0.16	0.57	0.15	1.51	0.03	0.49
p__Acidobacteria	0.55	0.30	0.41	0.47	0.10	0.26	0.09	0.09	0.93	0.31
c__Acidobacteria	0.55	0.30	0.41	0.47	0.10	0.26	0.09	0.09	0.93	0.31
o__Subgroup	0.55	0.30	0.41	0.47	0.10	0.26	0.09	0.09	0.93	0.31
f__uncultured	0.55	0.30	0.41	0.47	0.10	0.26	0.09	0.09	0.93	0.31
g__uncultured	0.55	0.30	0.41	0.47	0.10	0.26	0.09	0.09	0.93	0.31
p__Gemmatimonadetes	0.56	0.10	0.18	0.52	0.09	0.19	0.10	0.12	0.87	0.40
c__Gemmatimonadetes	0.56	0.10	0.18	0.52	0.09	0.19	0.10	0.12	0.87	0.40
o__Gemmatimonadales	0.52	0.08	0.22	0.50	0.09	0.21	0.08	0.11	0.71	0.43
f__Gemmatimonadaceae	0.52	0.07	0.20	0.50	0.09	0.21	0.08	0.11	0.71	0.43
g__Gemmatimonas	0.52	0.07	0.20	0.50	0.09	0.21	0.08	0.11	0.71	0.43
p__Cyanobacteria	0.17	0.06	0.41	0.23	0.07	0.15	0.06	0.18	0.16	0.43
c__Melainabacteria	0.17	0.06	0.38	0.23	0.07	0.15	0.06	0.18	0.16	0.43
o__Gastranaerophilales	0.17	0.06	0.38	0.23	0.07	0.15	0.06	0.18	0.16	0.43
f__uncultured	0.15	0.06	0.41	0.21	0	0.05	0.06	0.18	0.16	0.45

g__uncultured	0.15	0.06	0.41	0.21	0	0.05	0.06	0.18	0.16	0.45
p__Tenericutes	0.09	0.07	0.69	0.15	0	0.24	0.15	0.21	0.50	0.62
c__Mollicutes	0.09	0.07	0.69	0.15	0	0.24	0.15	0.21	0.50	0.62
o__Mollicutes RF9	0.08	0.06	0.68	0.14	0	0.38	0.14	0.18	0.65	0.65
f__uncultured	0.08	0	0.25	0.14	0	0.38	0.12	0.14	0.83	0.72
p__Nitrospirae	0.32	0	0.01	0.24	0	0.17	0.08	0	0.34	0.34
c__Nitrospira	0.32	0	0.01	0.24	0	0.17	0.08	0	0.34	0.34
o__Nitrospirales	0.32	0	0.01	0.24	0	0.17	0.08	0	0.34	0.34
f__Nitrospirales Incertae Sedis	0.31	0	<0.01	0.19	0	0.16	0.08	0	0.34	0.27
g__Candidatus Methylomirabilis	1.35	0.20	0.11	0.19	0	0.16	0.08	0	0.34	0.26
p__Tenericutes	0.09	0.07	0.56	0.15	0	0.04	0.10	0.21	0.03	0.17
c__Mollicutes	0.09	0.07	0.56	0.15	0	0.04	0.10	0.21	0.03	0.17
o__Tenericutes	0.08	0.06	0.49	0.14	0	0.05	0.10	0.18	0.05	0.42
f__ uncultured	0.08	0	0.49	0.14	0	0.05	0.10	0.14	0.05	0.42
p__Nitrospirae	0.32	0	0.02	0.24	0	0.01	0.07	0	0.12	0.09
c__Nitrospira	0.32	0	0.02	0.24	0	0.01	0.07	0	0.12	0.09
o__Nitrospirales	0.32	0	0.02	0.24	0	0.01	0.07	0	0.12	0.09
f__Nitrospirales Incertae Sedis	0.30	0	0.03	0.19	0	0.02	0.07	0	0.12	0.10
g__Candidatus Methylomirabilis	0.30	0	0.03	0.19	0	0.02	0.07	0	0.12	0.10
