# Supplementary material for: The underlying microbial mechanism of epizootic rabbit enteropathy triggered by a low fiber diet
Source: Sci Rep. 2018 Aug 21;8:12489. doi: 10.1038/s41598-018-30178-2 (PMC6104036; doi:10.1038/s41598-018-30178-2)
Supplement: Supplementary file 3 — Supplementary Table 2 [file 41598_2018_30178_MOESM3_ESM.pdf]

The underlying microbial mechanism of epizootic rabbit enteropathy triggered by a low fiber diet

DingXing Jin[a*], HuaWei Zou[a*], SiQiang Liu[a], LiZhi Wang[a], Bai Xue[a], De Wu[a], Gang Tian[a], Jingyi Cai[a], TianHai Yan[b], ZhiSheng Wang[a] & QuanHui Peng[a]

[a] Institute of Animal Nutrition, Key Laboratory of Bovine Low-Carbon Farming and Safe Production, Sichuan Agricultural University, Ya'an, Sichuan, 625014, PR China

[b] Agri-Food and Biosciences Institute, Hillsborough, Co. Down BT26 6DR, United Kingdom

*This two authors contributed equally to this paper.

Correspondence and requests for materials should be addressed to Q.P. and Z.W. (email: pengquanhui@126.com , wangzs67@163.com)

Address: Animal nutrition institute, Xinkang road 46#, Yucheng, Ya'an, Sichuan province. Post code: 625014

Tel: +86 15283511570

Table S2. Quantitative real-time PCR (qPCR) analysis of total bacteria and other organisms in different parts of the digestive tract in normal and ERE rabbits.

	Stomach				Small intestine				Caecum			
	NS		ES		NI		EI		NC		EC	
	Pr[1]	Mean[2]	Pr	Mean	Pr	Mean	Pr	Mean	Pr	Mean	Pr	Mean
Total bacteria	6/6	5.78[a]	6/6	5.54[b]	6/6	7.59[a]	6/6	7.21[b]	6/6	9.89[a]	6/6	9.27[b]
Clostridium												
C. coccoides group	6/6	3.04[b]	6/6	3.51[a]	6/6	4.65[b]	5/6	5.58[a]	6/6	7.58	6/6	7.64
C. leptum group	5/6	3.47[b]	6/6	4.02[a]	6/6	5.28	5/6	5.49	5/6	6.35[b]	6/6	7.75[a]
C. spiroforme	4/6	2.48	4/6	2.47	3/6	3.99	4/6	4.14	4/6	5.09[b]	6/6	6.50[a]
C. perfringens	4/6	2.08[b]	6/6	3.54[a]	5/6	3.19[b]	6/6	5.21[a]	5/6	5.46[b]	6/6	7.28[a]
Bacteroides												
Bacteroides-Prevotella	6/6	4.37	6/6	4.47	6/6	6.24[b]	6/6	6.52[a]	6/6	8.30	6/6	8.44
B. fragilis	3/6	2.68[b]	5/6	3.71[a]	6/6	5.09[b]	6/6	6.39[a]	6/6	6.02[b]	6/6	7.67[a]
B. ovatus	5/6	3.26[b]	5/6	4.06[a]	5/6	4.64[b]	4/6	5.45[a]	5/6	5.96[b]	5/6	7.07[a]
B. stercoris	3/6	2.17[b]	5/6	2.86[a]	5/6	4.04[b]	5/6	4.91[a]	4/6	5.20[b]	5/6	6.37[a]
B. thetaiotaomicron	5/6	2.38[b]	6/6	3.41[a]	6/6	5.16[b]	6/6	5.67[a]	4/6	6.51[b]	6/6	7.82[a]
B. caccae	3/6	<2.0[b]	3/6	2.64[a]	4/6	3.56[b]	5/6	4.69[a]	4/6	5.74[b]	4/6	6.83[a]
B. vulgatus	3/6	<2.0[b]	4/6	<2.0[a]	3/6	3.00[b]	3/6	3.61[a]	4/6	5.32[b]	4/6	6.49[a]
B. eggerthii	2/6	<2.0	4/6	<2.0	3/6	2.42[b]	3/6	2.92[a]	3/6	4.32[b]	3/6	5.16[a]
B. uniformis	3/6	<2.0	3/6	<2.0	2/6	2.20[b]	2/6	2.62[a]	3/6	4.27[b]	3/6	5.24[a]
Escherichia coli.	5/6	3.24[b]	6/6	4.07[a]	6/6	4.61[b]	6/6	5.85[a]	6/6	6.05[b]	6/6	8.18[a]

Shigella	6/6	4.04^{a}	5/6	3.78^{b}	5/6	6.03^{b}	6/6	5.28^{a}	5/6	7.31	5/6	7.08
Akkermansia muciniphila	6/6	3.40^{b}	6/6	4.03^{a}	6/6	5.05^{b}	6/6	6.57^{a}	6/6	6.22^{b}	6/6	8.39^{a}
Enterobacter sakazakii	3/6	3.31^{b}	5/6	4.15^{a}	4/6	4.39^{b}	5/6	5.93^{a}	4/6	5.84	6/6	6.04
Sphingomonas paucimobilis	6/6	3.11^{b}	6/6	4.72^{a}	4/6	4.92	5/6	4.82	5/6	5.66	6/6	5.98
Alistipes group	6/6	4.04^{a}	6/6	3.51^{b}	5/6	5.86^{a}	5/6	5.17^{b}	6/6	8.47^{a}	5/6	7.14^{b}
Lactobacillus group	6/6	4.13^{a}	5/6	2.51^{b}	6/6	4.86^{a}	4/6	3.87^{b}	6/6	6.92^{a}	4/6	6.16^{b}
Bifidobacterium group	6/6	3.94^{a}	4/6	2.66^{b}	6/6	5.80^{a}	3/6	4.01^{b}	6/6	7.06^{a}	2/6	6.10^{b}
Butyrivibrio fibrisolvens	5/6	3.32^{a}	3/6	2.10^{b}	4/6	5.41^{a}	3/6	3.75^{b}	4/6	5.98^{a}	3/6	5.26^{b}
Eubacterium group	5/6	3.91^{a}	4/6	3.20^{b}	5/6	5.47^{a}	5/6	4.50^{b}	4/6	6.84^{a}	4/6	5.31^{b}
Faecalibacterium prausnitzii	4/6	3.66	4/6	3.52	6/6	5.10^{a}	4/6	3.69^{b}	3/6	7.29^{a}	4/6	6.02^{b}
Ruminococcus												
R. albus	6/6	3.68	4/6	3.54	6/6	5.59^{a}	5/6	5.21^{b}	5/6	7.89^{a}	5/6	6.27^{b}
R. flavefaciens	6/6	3.93^{a}	5/6	3.69^{b}	6/6	5.64	5/6	5.36	5/6	8.04^{a}	4/6	6.92^{b}

[1]Prevalence (Pr) reflects the percentage of positive amplifications from total samples analysed by PCR

[2]Data are presented as mean $\log_{10}$ $^{\text{copy number/g content}}$, n=6; means in normal groups (NS, NI and NC) with different superscript compared with ERE groups (ES, EI and EC) represent significant difference.
