# Supplementary material for: The underlying microbial mechanism of epizootic rabbit enteropathy triggered by a low fiber diet
Source: Sci Rep. 2018 Aug 21;8:12489. doi: 10.1038/s41598-018-30178-2 (PMC6104036; doi:10.1038/s41598-018-30178-2)
Supplement: Supplementary file 4 — Supplementary Table 3 [file 41598_2018_30178_MOESM4_ESM.pdf]

The underlying microbial mechanism of epizootic rabbit enteropathy triggered by a low fiber diet

DingXing Jin[a*], HuaWei Zou[a*], SiQiang Liu[a], LiZhi Wang[a], Bai Xue[a], De Wu[a], Gang Tian[a], Jingyi Cai[a], TianHai Yan[b], ZhiSheng Wang[a] & QuanHui Peng[a]

[a] Institute of Animal Nutrition, Key Laboratory of Bovine Low-Carbon Farming and Safe Production, Sichuan Agricultural University, Ya'an, Sichuan, 625014, PR China

[b] Agri-Food and Biosciences Institute, Hillsborough, Co. Down BT26 6DR, United Kingdom

*This two authors contributed equally to this paper.

Correspondence and requests for materials should be addressed to Q.P. and Z.W. (email: pengquanhui@126.com , wangzs67@163.com)

Address: Animal nutrition institute, Xinkang road 46#, Yucheng, Ya'an, Sichuan province. Post code: 625014

Tel: +86 15283511570

Table S3. Species-specific 16S-targeted primers and optimized conditions for qPCR

Genus	Target organism(s)	Sequence (5`–3`)	Annealing temperature (℃)	Reference
Total bacteria		F: ACTCCTACGGGAGGCAGCAG<br>R: ATTACCGCGGCTGCTGG	55	52
Clostridium	*C. coccoides* group	F: AAATGACGGTACCTGACTAA<br>R: CTTTGAGTTTCATTCTTGCGAA	50	53
	C. leptum group	F: GCACAAGCAGTGGAGT<br>R: CTTCCTCCGTTTTGTCAA	50	53
	C. spiroforme	F：TTGAGCGATTTACTTCGGTAAAGA<br>R: CCATCCTGTACTGGCTCACCT	58	54
	C. perfringens	F：ATGCAAGTCGAGCGAGG<br>R：TATGCGGTATTAATCTCCCTTT	55	54
Bacteroides	*Bacteroides-Prevotella*	F: ATAGCCTTTCGAAAGRAAGAT<br>R: CCAGTATCAACTGCAATTTTA	50	53
	B. vulgatus	F: GCATCATGAGTCCGCATGTTC<br>R: TCCATACCCGACTTTATTCCTT	63	55
	B. fragilis	F: TCRGGAAGAAAGCTTGCT<br>R: CATCCTTTACCGGAATCCT	63	
	B. ovatus	F: TGCAAACTRAAGATGGC<br>R: CAAACTAATGGAACGCATC	58	
	B. stercoris	F: GCTTGCTTTGATGGATGGC<br>R: CATGCGGGAAAACTATGCC	63	

	B. thetaiotaomicron	F: GCAAACTGGAGATGGCGA<br>R: AAGGTTTGGTGAGCCGTTA	62.5	
	B. caccae	F: AAACCCATACGCCGCAAG<br>R: GACACCTCACGGCACGAG	63	
	B. eggerthii	F: CCCGATAGTATAGTTTTTCCGC<br>R: TCCTCTCAGAACCCCTATCCAT	62	
	B. uniformis	F: TCTTCCGCATGGTAGAACTATTA<br>R: ACCGTGTCTCAGTTCCAATGTG	60	
Escherichia	*E. coli*	F: GTTAATACCTTTGCTCATTGA<br>R: ACCAGGGTATCTAATCCTGTT	61	56
Shigella	*Shigella*	F: TGAAGTTTCTCTGCGAGCAT<br>R: TGAAGTTTCTCTGCGAGCAT	59	57
Akkermansia	*A. muciniphila*	F: CAG CAC GTG AAG GTG GGG AC<br>R: CCT TGC GGT TGG CTT CAG A	50	58
Enterobacter	*E. sakazakii*	F: TATAGGTTGTCTGCGAAAGCG<br>R: GTCTTCGTGCTGCGAGTTT	59	59
Sphingomonas	*S.paucimobilis*	F: AACTGGAGGAAGGTGGGGAT<br>R: AGGAGGTGATCCAACCGCA	60	60
Alistipes	*Alistipes* group	F: TTAGAGATGGGCATGCGTTGT<br>R: TGAATCCTCCGTATTACCGCG	60	61
Lactobacillus	*Lactobacillus* group	F: AGCAGTAGGGAATCTTCCA<br>R: CACCGCTACACATGGAG	58	54
Bifidobacterium	*Bifidobacterium* group	F: GCGTGCTTAACACATGCAAGTC<br>R: CACCCGTTTCCAGGAGCTATT	59	62
Butyrivibrio	*B. fibrisolvens*	F: ACACACCGCCCGTCACA<br>R: TCCTTACGGTTGGGTCACAGA	59	63
Eubacterium	*Eubacterium* group	F: GCTTCTGAAGAATCATTTGAAG<br>R: TCGTGCCTCAGTGTCAGTGT	57	64
Faecalibacterium	*F. prausnitzii*	F: CCCTTCAGTGCCGCAGT<br>R: GTCGCAGGATGTCAAGAC	61	54
Ruminococcus	*R. albus*	F: CCCTAAAAGCAGTCTTAGTTCG<br>R: CCTCCTTGCGGTTAGAACA	60	65
	R. flavefaciens	F: TCTGGAAACGGATGGTA<br>R: CCTTTAAGACAGGAGTTTACAA	60	

52. Han, G. Q. et al. Effects of different starch sources on Bacillus spp. in intestinal tract and expression of intestinal development related genes of weanling piglets. *Molecular biology reports* **39**, 1869-1876 (2012).
53. Matsuki, T. et al. Development of 16S rRNA-gene-targeted group-specific primers for the detection and identification of predominant bacteria in human feces. *Applied and environmental microbiology* **68**, 5445-5451 (2002).
54. Rinttilä, T. et al. Development of an extensive set of 16S rDNA-targeted primers for quantification of pathogenic and indigenous bacteria in faecal samples by real-time PCR. *Journal of applied microbiology* **97**, 1166-1177 (2004).
55. Tong, J. et al. Application of quantitative real-time PCR for rapid identification of Bacteroides fragilis group and related organisms in human wound samples. *Anaerobe* **17**, 64-68 (2011).
56. Malinen, E. et al. Analysis of the fecal microbiota of irritable bowel syndrome patients and healthy controls with real-time PCR. *The American journal of gastroenterology* **100**, 373-382 (2005).
57. Buchan, B. W et al. Clinical evaluation of a real-time PCR assay for identification of Salmonella, Shigella, Campylobacter (Campylobacter jejuni and C. coli), and shiga toxin-producing Escherichia coli isolates in stool specimens. *Journal of Clinical Microbiology* **51**, 4001-4007 (2013).
58. Collado, M. C. et al. Intestinal integrity and Akkermansia muciniphila, a mucin-degrading member of the intestinal microbiota present in infants, adults, and the elderly. *Applied and environmental microbiology* **73**, 7767-7770 (2007).
59. Liu, Y. et al. Real time PCR using TaqMan and SYBR Green for detection of Enterobacter sakazakii in infant formula. *Journal of Microbiological Methods* **65**,21-31 (2006).
60. Joseph, C. R. et al. Real-time Polymerase Chain Reaction in the Diagnosis of Acute Postoperative Endophthalmitis. *American Journal of Ophthalmology* **153**, 1031-1037 (2012)
61. Vigsnæs, L. K. et al. Gram-negative bacteria account for main differences between faecal microbiota from patients with ulcerative colitis and healthy controls. *Beneficial Microbes* **3**, 287-297 (2012).
62. Penders, J. et al. Quantification of *Bifidobacterium* spp., *Escherichia coli* and *Clostridium difficile* in faecal samples of breast-fed and formula-fed infants by real-time PCR. *FEMS microbiology letters* 243, 141-147 (2005).
63. Klieve, A. V. et al. Establishing populations of *Megasphaera elsdenii* YE 34 and *Butyrivibrio fibrisolvens* YE 44 in the rumen of cattle fed high grain diets. *Journal of Applied Microbiology* **95**, 621-630 (2003).
64. Tajima, K. et al. Diet-Dependent Shifts in the Bacterial Population of the Rumen Revealed with Real-Time PCR. *Applied and Environmental Microbiology* **67**, 2766-2774 (2001).
65. Koike, S. & Kobayashi, Y. Development and use of competitive PCR assays for the ruminal cellulolytic bacteria: *Fibrobacter succinogenes*, *Ruminococcus albus* and *Ruminococcus flavefaciens*. *FEMS Microbiol Letter* **204**, 361-366 (2001).
